# Alzheimer’s genetic risk factor CASS4 controls microglial focal adhesion biology and amyloidosis

**DOI:** 10.1084/jem.20260104

**Published:** 2026-10-27

**Authors:** Adeline E. Walsh, Benjamin A. Davis, Joshua D. Samuels, William A. Mills, Jessica M. Thanos, Alexis M. Johnson, Katelyn A. Moore, Michael A. Puchalski, Eric J. Steacy, Benjamin W. Wendell, Amélie Daugherty-Lopès, Adrian A. Shimpi, Ukpong B. Eyo, John R. Lukens

**Affiliations:** 1 Center for Brain Immunology and Glia (BIG), Harrison Family Translational Research Center in Alzheimer’s and Neurodegenerative Diseases, Department of Neuroscience, University of Virginia, Charlottesville, VA, USA; 2 Physiology Graduate Program, University of Virginia, Charlottesville, VA, USA; 3 Neuroscience Graduate Program, University of Virginia, Charlottesville, VA, USA; 4 Department of Biomedical Engineering, University of Virginia, Charlottesville, VA, USA

## Abstract

Recent genome-wide association studies have identified numerous Alzheimer’s disease (AD) risk genes enriched in microglia, including Crk-associated substrate (Cas) scaffold protein family member 4 (*CASS4*), which encodes the adaptor protein CASS4. Despite its strong genetic association with AD, the role of CASS4 in disease remains poorly understood. Here, we show that full-body *Cass4* deletion in 5xFAD mice increases amyloid-β (Aβ) deposition, neuronal dystrophy, and cognitive deficits. Since microglia are the predominant brain-resident cells expressing *Cass4*, we investigated its role in microglial responses to Aβ pathology. Loss of CASS4 impaired microglial mobilization to Aβ plaques and reduced plaque compaction. Phosphoproteomic analysis of purified microglia revealed marked disruption of focal adhesion signaling following *Cass4* deletion. Consistent with this, CASS4 deficiency abrogated in vivo microglial recruitment to injury and in vitro wound healing responses, two processes that are centrally orchestrated by focal adhesion biology. Together, these findings identify CASS4 as a critical regulator of microglial function and focal adhesion biology and suggest that CASS4 may represent a therapeutic target for AD.

## Teaser

CASS4 is a genetic risk factor for Alzheimer’s disease but remains understudied in the brain. This study identifies CASS4 as a critical regulator of amyloid β plaque compaction, microglial mobilization to damage, and focal adhesion signaling.

## Introduction

Alzheimer’s disease (AD) is a neurodegenerative condition that currently impacts over six million Americans and is the sixth leading cause of death. Although AD-induced dementia and memory loss are classically accompanied by the deposition of amyloid β (Aβ) plaques and neurofibrillary tau tangles, emerging genome-wide association studies (GWAS) and subsequent meta-analyses point toward AD pathogenesis being much more complex (EADB et al., 2026; Lambert et al., 2013; Lin et al., 2017; Ramos de Matos et al., 2018; Schwartzentruber et al., 2021; Wang et al., 2015b). Most notably, these genetic studies implicate central roles for the innate immune system, with many AD risk genes being primarily expressed in microglia (Efthymiou and Goate, 2017; Karch and Goate, 2015; Lopes et al., 2022).

Microglia are the brain’s resident immune cells that use their motile processes to surveil the parenchyma for signs of infection, damage, or disease (Ennerfelt and Lukens, 2020). Studies conducted in AD mouse models have solidified pivotal roles for microglia in AD-related pathogenesis (Butovsky et al., 2026; Condello et al., 2015; Hansen et al., 2018; Heneka et al., 2025; Reed-Geaghan et al., 2020; Sarlus and Heneka, 2017; Spangenberg et al., 2016; Spangenberg et al., 2019; Tejera and Heneka, 2019). While these studies clearly define microglia as important players in AD, we are only just beginning to fully appreciate the molecular and effector mechanisms employed by microglia to influence neurodegenerative disease. Microglia are deployed to form stable, protective barriers around Aβ plaques in the AD brain, and work to compact Aβ into tightly packed, less toxic forms (Casali et al., 2020; Condello et al., 2015; Zhao et al., 2017). These protective functions of microglia have been shown to limit axonal dystrophy to neurons in the immediate area, with the degree of plaque compaction positively correlating with neuronal health (Casali et al., 2020; Condello et al., 2015; Zhao et al., 2017). In contrast, chronic activation of microglia can also increase neuronal loss and drive disease progression through the release of harmful inflammatory cytokines (Wang et al., 2015a) and activation of astrocytes (Liddelow et al., 2017).

While significant progress has been made in defining roles for several of the recently identified AD genetic risk factors, many GWAS-identified genes remain understudied and poorly understood. Among these genes, Crk-associated substrate (Cas) scaffold protein family member 4 (*CASS4*) has been consistently associated with an increased risk of developing AD in human genetics studies and is also selectively expressed by microglia in the central nervous system (CNS) (Gosselin et al., 2017; Karlsson et al., 2021; Lambert et al., 2013; Lin et al., 2017; Podlesny-Drabiniok et al., 2020; Ramos de Matos et al., 2018; Schwartzentruber et al., 2021; Wang et al., 2015b; Zhang et al., 2014). Despite *CASS4* being increasingly linked to AD risk in humans (EADB et al., 2026; Lambert et al., 2013; Lin et al., 2017; Ramos de Matos et al., 2018; Schwartzentruber et al., 2021; Wang et al., 2015b), little is currently known about CASS4 biology or its role in neurological health and disease. What is known about CASS4 largely comes from a handful of studies exploring its biology in cell lines and cancer (Barrett et al., 2013; Deneka et al., 2015; Tikhmyanova et al., 2010). These studies have helped to identify CASS4 as a member of the Cas protein family (Singh et al., 2008). Members of the Cas family lack enzymatic activity and instead act as scaffold proteins with numerous protein-binding domains. These domains serve as assembly sites for large protein complexes. Cas proteins are best known for their critical involvement in the regulation of focal adhesions (Deneka et al., 2015; Singh et al., 2008), which are dynamic, actin-containing cytoskeletal structures that endow cells with the membrane protrusions and traction forces needed to migrate, sculpt extracellular structures, and phagocytose material (Geiger et al., 2009; Wozniak et al., 2004).

To break new ground in our understanding of how CASS4 impacts AD pathogenesis, we generated *Cass4* knockout mice and explored the role of *Cass4* deletion in the 5xFAD mouse model of AD-related amyloidosis. Leveraging this new genetic tool, we found that loss of CASS4 in 5xFAD mice leads to increased Aβ plaque burden, exacerbated neuritic dystrophy, and impaired cognitive flexibility. Aggravated amyloidosis in CASS4-deficient 5xFAD mice was further revealed to be associated with defects in the ability of microglia to engage with Aβ, as well as defective plaque compaction. In line with a microglia-intrinsic role for CASS4 in amyloidosis, we also observed increased Aβ plaque load, worsened neuritic dystrophy, and defective plaque compaction with microglia-targeted *Cass4* deletion. Phosphoproteomic array analysis of purified microglia demonstrated that deletion of *Cass4* disrupted focal adhesion signaling in AD-associated amyloidosis. Consistent with these signaling findings, we also observed that the loss of CASS4 leads to impaired in vivo microglial recruitment to laser injury, as well as compromised in vitro wound-healing responses, both of which are processes governed by focal adhesion mechanisms. Taken together, these findings define novel roles for CASS4 in AD progression, microglial function, and the regulation of focal adhesion–dependent pathways and suggest that CASS4 represents a potential therapeutic target for AD.

## Results

### Loss of *Cass4* in 5xFAD mice results in increased Aβ burden

**Figure S1.**
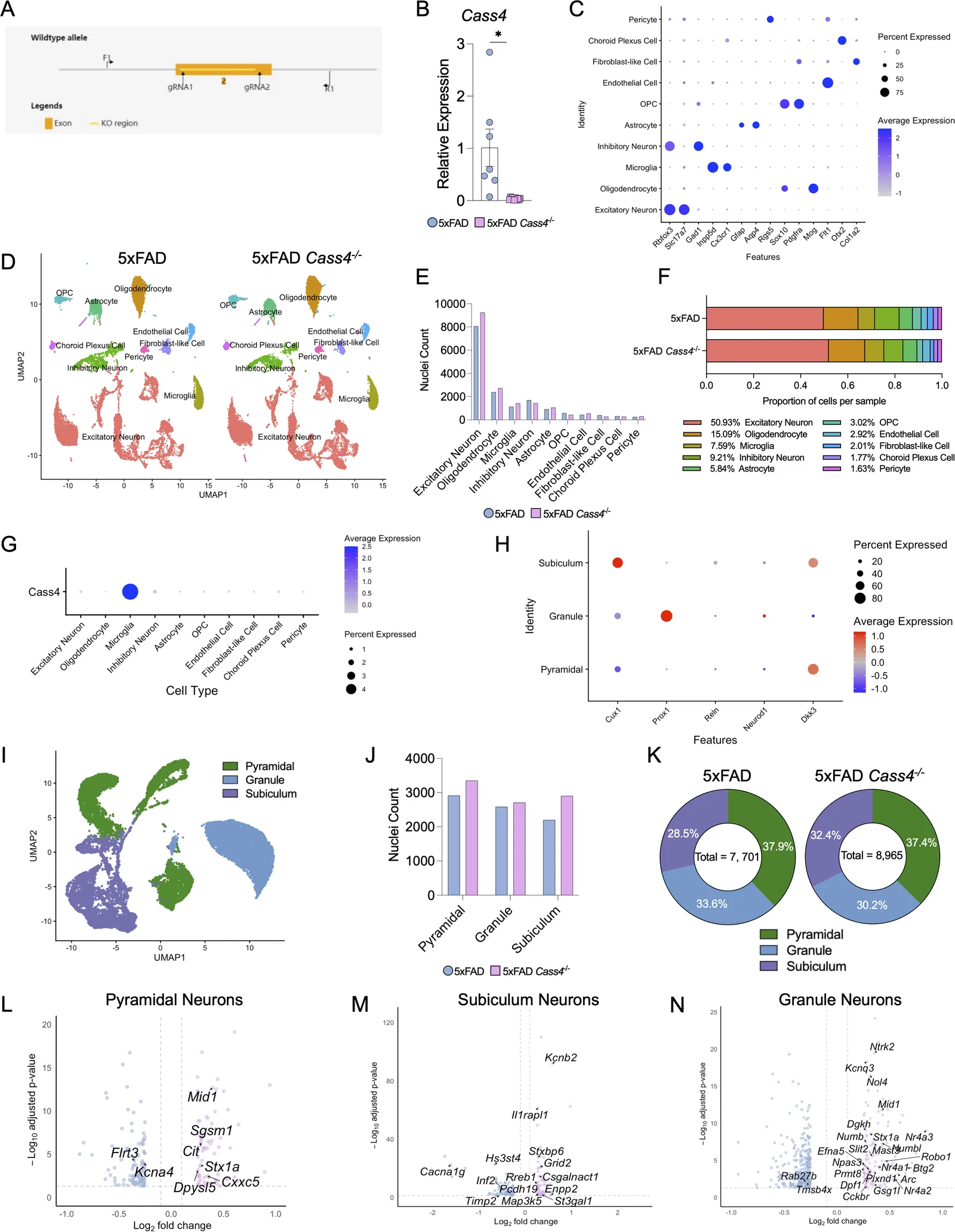
**snRNA-seq analysis of major hippocampal cell types in 5xFAD *Cass4*** ^**−/−**^ **mice. (A)** Gene targeting strategy to generate *Cass4*^−/−^ mice. **(B)** Relative expression of *Cass4* in whole brain samples of 5xFAD and 5xFAD Cass4^−/−^ mice measured by qPCR at 5 mo of age. Statistics for qPCR were calculated with a Welch’s *t* test, and each data point represents an individual mouse. Error bars represent mean ± SEM. *P < 0.05. **(C–G)** snRNA-seq was performed on pooled hippocampal tissue from 5xFAD *Cass4*^*−/−*^ mice and 5xFAD littermate controls harvested at 5 mo of age (*n* = 6 mice/group). **(C)** Dot plot depicting the nuclei identity of each cluster, as determined by the expression of cell type–specific markers. **(D)** UMAP depicting 33,985 nuclei isolated from 5xFAD *Cass4*^*−/−*^ mice (17,797 total nuclei) and 5xFAD samples (16,188 total nuclei) showing 10 distinct brain cell types. **(E)** Bar chart showing the nuclei distribution per cell type in each group. **(F)** Bar chart showing the relative nuclei distribution across all cell types in each group. **(G)** Dot plot of *Cass4* expression by cell type in all samples. **(H–N)** The excitatory neuron cluster was further subclustered. **(H)** Dot plot depicting the nuclei identity of each subcluster, as determined by the expression of cell type–specific markers. **(I)** UMAP plot depicting three unique excitatory neuron subclusters. **(J)** Bar chart showing the nuclei distribution per cell type in each group. **(K)** Donut chart depicting the frequency of nuclei recovered per cell type across both groups. **(L)** Volcano plot depicting DEGs (upregulated, pink; downregulated, blue) in the pyramidal excitatory neuron cluster of 5xFAD *Cass4*^−/−^ versus 5xFAD hippocampus. Colored points depict genes with adjusted P values <0.05 and log_2_ fold changes ±0.1. **(M)** Volcano plot depicting DEGs (upregulated, pink; downregulated, blue) in the subiculum excitatory neuron cluster of 5xFAD *Cass4*^−/−^ versus 5xFAD hippocampus. Colored points depict genes with adjusted P values <0.05 and log_2_ fold changes ±0.1. **(N)** Volcano plot depicting DEGs (upregulated, pink; downregulated, blue) in the granule excitatory neuron cluster of 5xFAD *Cass4*^−/−^ versus 5xFAD hippocampus. Colored points depict genes with adjusted P values <0.05 and log_2_ fold changes ±0.1.

**Figure 1.**
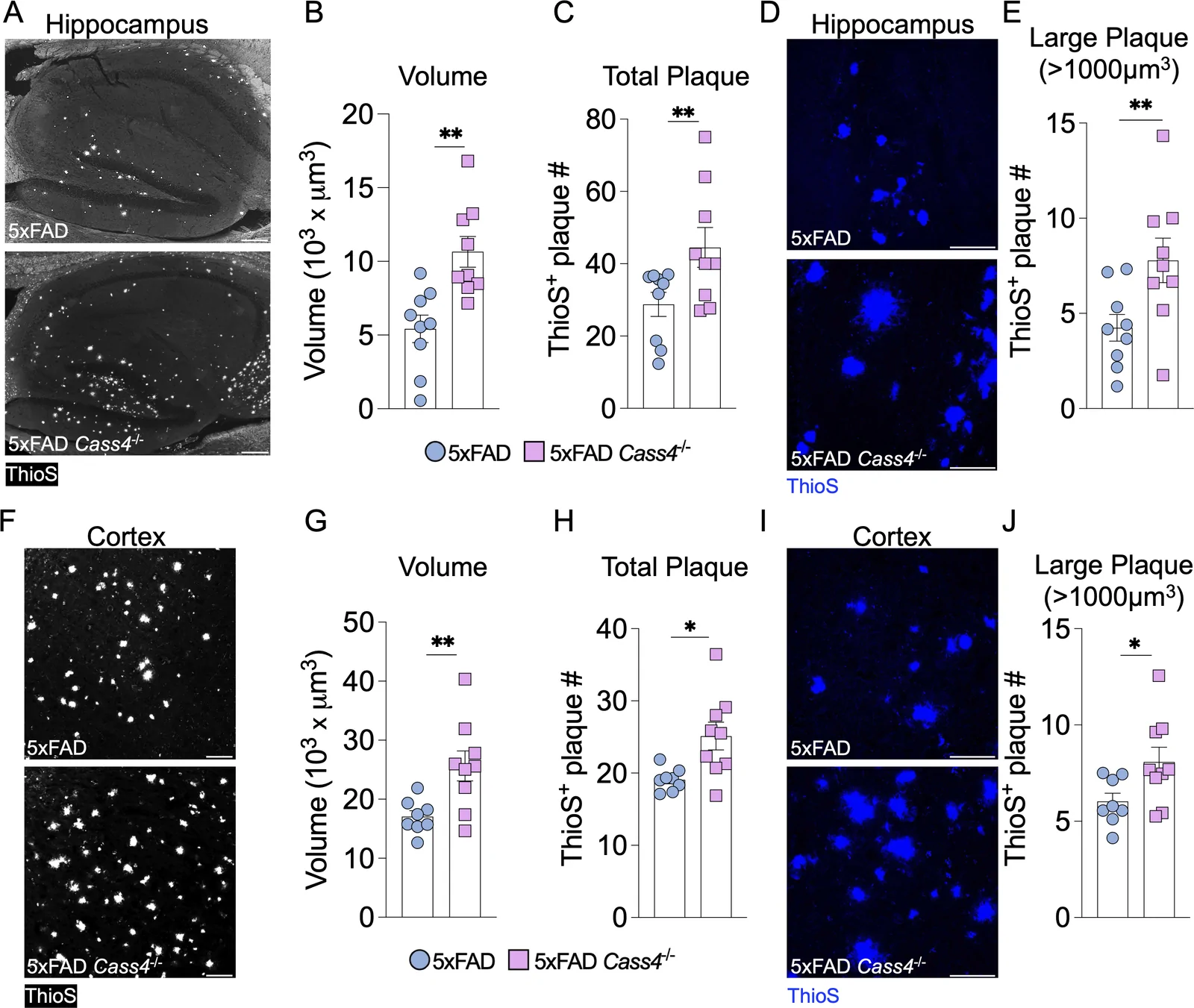
**Genetic ablation of *Cass4* in 5xFAD mice leads to increased Aβ load.** Brains from 5xFAD *Cass4*^−/−^ mice and 5xFAD littermate controls were harvested at 5 mo of age to evaluate Aβ load by confocal imaging. **(A)** Representative tile scans of the hippocampus with ThioS staining (Aβ plaques, white) taken at 20× magnification. Scale bar: 100 μm. **(B)** Total ThioS^+^ Aβ plaque volume per field of view (FOV) in the hippocampus. **(C)** ThioS^+^ Aβ plaque number per FOV in the hippocampus. **(D)** Representative images of Aβ plaque in the dentate gyrus region of the hippocampus (ThioS, blue) taken at 63× magnification. Scale bar: 40 μm. **(E)** Large plaque number, as determined by a ThioS volume of >1,000 μm^3^ per Aβ plaque in the hippocampus. **(F)** Representative tile scans of the cortex with ThioS staining (Aβ plaques, white) taken at 20× magnification. Scale bar: 100 μm. **(G)** Total ThioS^+^ Aβ plaque volume per FOV in the cortex. **(H)** ThioS^+^ Aβ plaque number per FOV in the cortex. **(I)** Representative images of Aβ plaques in the cortex (ThioS, blue) taken at 63× magnification. Scale bar: 40 μm. **(J)** Large plaque number, as determined by a ThioS volume of >1,000 μm^3^ per Aβ plaque in the cortex. For all imaging experiments, three brain sections per mouse were imaged, with at least three FOV taken per section per region (a total of 9–12 images per mouse). Statistical significance between experimental groups was calculated by a linear mixed-effects model. *P < 0.05, **P < 0.01. Error bars represent mean ± SEM, and each data point represents an individual mouse.

To interrogate the involvement of CASS4 in Aβ-mediated neuropathology, we generated germline *Cass4* knockout mice and crossed them with 5xFAD mice (hereafter referred to as 5xFAD *Cass4*^*−/−*^ mice), which is a widely used AD mouse model of amyloidosis (Oakley et al., 2006; Fig. S1, A and B). 5xFAD *Cass4*^*+/+*^ littermates (hereafter referred to as 5xFAD mice) were used as controls in our studies. Leveraging this newly generated 5xFAD *Cass4*^*−/−*^ mouse line, we first sought to characterize Aβ load in the hippocampus (dentate gyrus and CA1 regions) and cortex of 5-mo-old mice. Using Thioflavin S (ThioS) to label Aβ plaques, we found that 5xFAD *Cass4*^*−/−*^ mice exhibited a greater total volume of ThioS^+^ staining and increased numbers of ThioS^+^ Aβ plaques in the hippocampus relative to 5xFAD littermate controls (Fig. 1, A–C). 5xFAD *Cass4*^*−/−*^ mice also had elevated numbers of large plaques in the hippocampus, which were defined as a plaque >1,000 μm^3^ in volume (Fig. 1, D and E). Similar to the hippocampus, we observed a greater total volume of ThioS^+^ staining and increased numbers of ThioS^+^ Aβ plaques in the cortex of 5xFAD *Cass4*^*−/−*^ mice (Fig. 1, F–H), in addition to a greater number of large plaques (Fig. 1, I and J). These data suggest that deletion of *Cass4* leads to increased Aβ deposition in the brain.

### Neuronal health is impaired in 5xFAD *Cass4*^*−/−*^ mice

**Figure 2.**
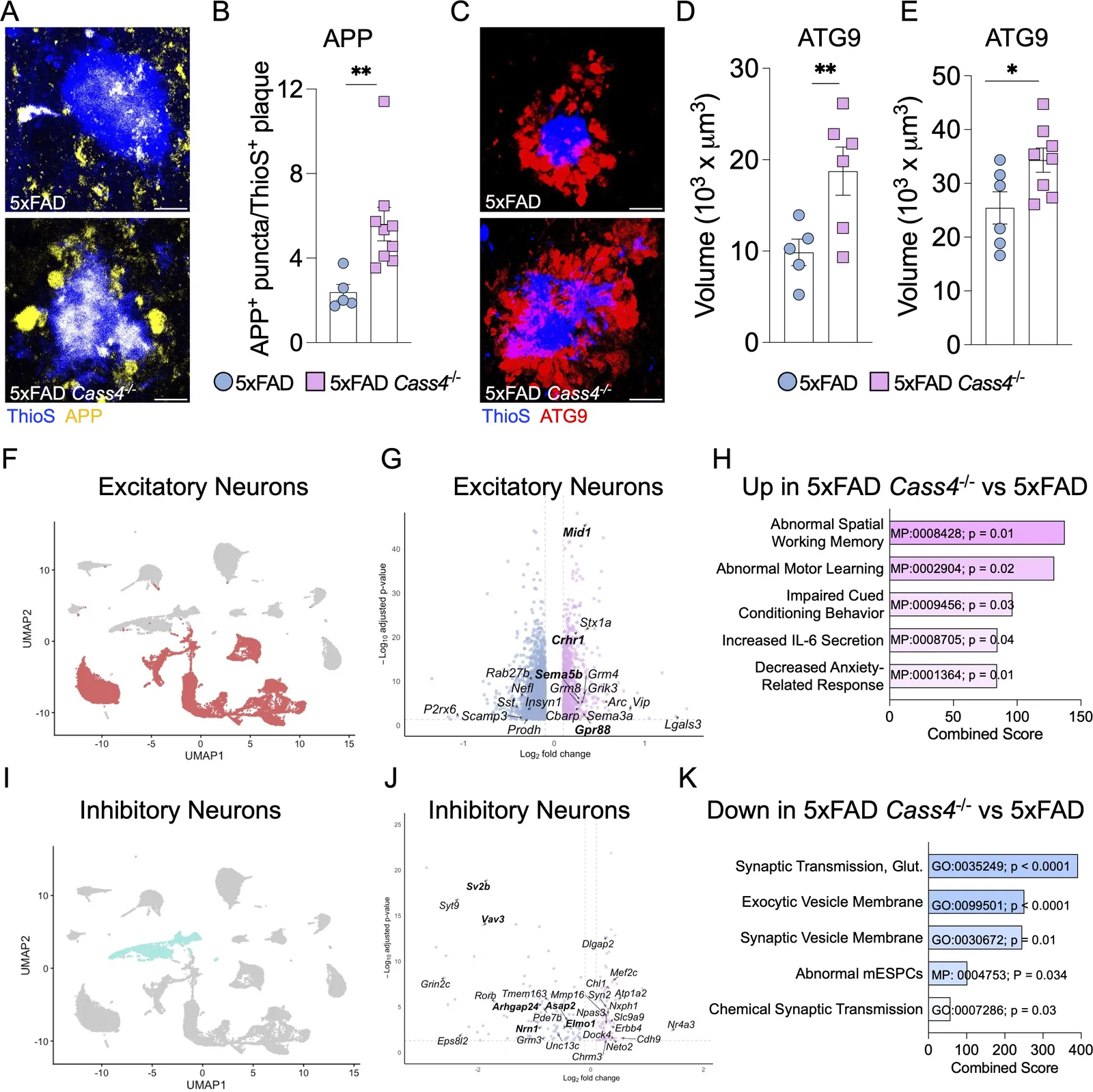
**Loss of *Cass4* in 5xFAD mice results in worsened neuronal health.** Brains from 5xFAD *Cass4*^−/−^ mice and 5xFAD littermate controls were harvested at 5 mo of age to evaluate neuronal health by confocal imaging. **(A)** Representative images for dystrophic neurites (APP, yellow) and Aβ plaques (ThioS, blue) in the hippocampus at 63× magnification. Scale bar: 7 μm. **(B)** Quantification of APP^+^ puncta within 30 μm of an Aβ plaque in the hippocampus per FOV. **(C)** Representative images for dystrophic neurites (ATG9, red) and Aβ plaques (ThioS, blue) in the hippocampus at 63× magnification. Scale bar: 7 μm. **(D)** Total volume of ATG9^+^ puncta in the hippocampus per FOV. **(E)** Total volume of ATG9^+^ puncta in the cortex per FOV. **(F–K)** For snRNA-seq, hippocampi from 5xFAD *Cass4*^−/−^ mice and 5xFAD littermate controls were micro-dissected at 5 mo of age and pooled (*n* = 6 mice/group). **(F)** UMAP plot displaying the excitatory neuron cluster. **(G)** Volcano plot depicting DEGs (upregulated, pink; downregulated, blue) in the excitatory neuron cluster of 5xFAD *Cass4*^−/−^ versus 5xFAD. Colored points depict genes with adjusted P values <0.05 and log_2_ fold changes ±0.1. **(H)** Bar plots depicting upregulated mammalian phenotype (MP) terms enriched in 5xFAD *Cass4*^*−/−*^ excitatory neurons. **(I)** UMAP plot depicting the inhibitory neuron cluster. **(J)** Volcano plot depicting DEGs (upregulated, pink; downregulated, blue) in the inhibitory neuron cluster of 5xFAD *Cass4*^−/−^ versus 5xFAD. Colored points depict genes with adjusted P values <0.05 and log_2_ fold changes ±0.1. **(K)** Bar plots depicting downregulated MP and Gene Ontology (GO) terms enriched in 5xFAD *Cass4*^*−/−*^ inhibitory neurons. Functional enrichment analysis was performed with g:Profiler using genes with adjusted P values <0.05 and log_2_ fold changes ±0.25. Depicted pathways were curated from the top driver terms in MP and GO. For all imaging experiments, three brain sections per mouse were imaged, with at least three FOV taken per section per region (a total of 9–12 images per mouse). Statistics for imaging experiments were calculated by linear mixed-effects model *P < 0.05, **P < 0.01. Error bars represent mean ± SEM, and each data point represents an individual mouse.

Given the worsened Aβ pathology seen in *Cass4*-deficient 5xFAD mice (Fig. 1), we were next interested in exploring how loss of CASS4 impacts neuronal health in AD-associated amyloidosis. The buildup of amyloid precursor protein (APP) puncta is commonly used as a marker of swollen and damaged neuronal processes. We observed an increase in APP^+^ dystrophic neurites per plaque in 5xFAD *Cass4*^*−/−*^ mice when compared with 5xFAD littermate controls in the hippocampus (Fig. 2, A and B). The mislocalization of transmembrane autophagy-related protein 9A (ATG9) to axons has also been used to characterize unhealthy, dystrophic neurites. In line with our APP puncta data, we found that there was elevated ATG9 staining in the hippocampus and cortex of 5xFAD *Cass4*^*−/−*^ mice relative to 5xFAD littermate controls (Fig. 2, C–E). These collective findings point toward a greater level of neuronal dystrophy upon loss of CASS4 in 5xFAD mice.

To further investigate how *Cass4* deletion affects AD-related amyloidosis, we next performed single-nucleus RNA sequencing (snRNA-seq) on flash-frozen, pooled, full hippocampi (*n* = 6 per genotype) from 5-mo-old 5xFAD and 5xFAD *Cass4*^*−/−*^ mice (Fig. S1, C–G). Canonical gene markers were used to identify brain major cell types, including excitatory neurons (*Slc17a7*), inhibitory neurons (*Gad1*), oligodendrocytes (*Mog*), astrocytes (*Gfap*, *Aqp4*), microglia (*Inpp5d*, *Cx3cr1*), oligodendrocyte precursor cells (*Pdgfra*), endothelial cells (*Flt1*), fibroblasts (*Col1a2*), pericytes (*Rgs5*), and choroid plexus cells (*Otx2*) (Fig. S1 C). Nuclei across both genotypes were visualized by uniform manifold approximation and projection (UMAP), and unsupervised clustering of 33,985 nuclei identified 10 transcriptionally distinct populations (Fig. S1 D). Analysis of cell type numbers showed similar proportions of major populations between 5xFAD and 5xFAD *Cass4*^*−/−*^ samples, indicating that *Cass4* deletion does not produce large-scale shifts in hippocampal cell composition (Fig. S1, E and F). As previously reported, we found that *Cass4* is predominantly expressed by microglia in the brain (Fig. S1 G; Lee et al., 2023; Podlesny-Drabiniok et al., 2020).

Since excitatory neurons are especially affected in AD patients (Arriagada et al., 2026; Basha et al., 2026; Bhembre et al., 2023; Burns et al., 2026; Gabitto et al., 2024), we first examined how *Cass4* deletion impacts their gene expression in the hippocampus. In the excitatory neuron cluster (Fig. 2 F), we identified 2,982 differentially expressed genes (DEGs) between 5xFAD and 5xFAD *Cass4*^−/−^ excitatory neurons (Fig. 2 G). Furthermore, we found that 5xFAD *Cass4*^*−/−*^ excitatory neurons exhibited an upregulation of genes and pathways related to spatial working memory and motor learning such as *Mid1*, *Gpr88*, *Crhr1*, and *Sema5b* (Galet et al., 2021; Heinz et al., 2021; Liu et al., 2014; Lv et al., 2024; Ma et al., 2025; Matthes et al., 2018; Meirsman et al., 2016; O’Connor et al., 2009; Fig. 2, G and H). We next further subclustered the excitatory neuron population into pyramidal neurons, subiculum neurons, and granule neurons to gain deeper insights into which subpopulation was most greatly affected in 5xFAD *Cass4*^−/−^ mice (Fig. S1, H and I). Here, we identified relatively equivalent numbers and percentages of pyramidal, granule, and subiculum excitatory neurons between 5xFAD *Cass4*^*−/−*^ and 5xFAD mice (Fig. S1, J and K). Moreover, we found that the excitatory neuron DEGs were spread across each of the subpopulations (Fig. S1, L–N).

Inhibitory neurons are also known to play key roles in AD (Burns et al., 2026; Gabitto et al., 2024; Kurucu et al., 2022; Xu et al., 2020). Therefore, we next explored the impact of *Cass4* deletion on gene expression in 5xFAD *Cass4*^−/−^ mice. Here, we identified 213 DEGs between inhibitory neurons isolated from 5xFAD *Cass4*^−/−^ and 5xFAD mice (Fig. 2, I and J). Moreover, we observed a downregulation of genes and pathways involved in neuronal circuitry such as *Sv2b*, *Vav3*, *Arhgap24*, *Asap2*, *Elmo1*, and *Nrn1* in 5xFAD *Cass4*^−/−^ mice relative to 5xFAD littermate controls (Agrawal et al., 2026; Chan et al., 2020; Cowan et al., 2005; Falace et al., 2024; Hale et al., 2011; Kim et al., 2011; Li et al., 2018; Miyamoto et al., 2020; Pugh et al., 2026; Sauzeau et al., 2010; Stout et al., 2019; Yoon et al., 2025; Fig. 2, J and K). Taken together, these findings suggest that *Cass4* deletion leads to exacerbated neuritic dystrophy and increased expression of genes associated with memory and learning in the hippocampus of 5xFAD *Cass4*^−/−^ mice.

### *Cass4* deletion in 5xFAD mice leads to defects in cognitive flexibility

**Figure 3.**
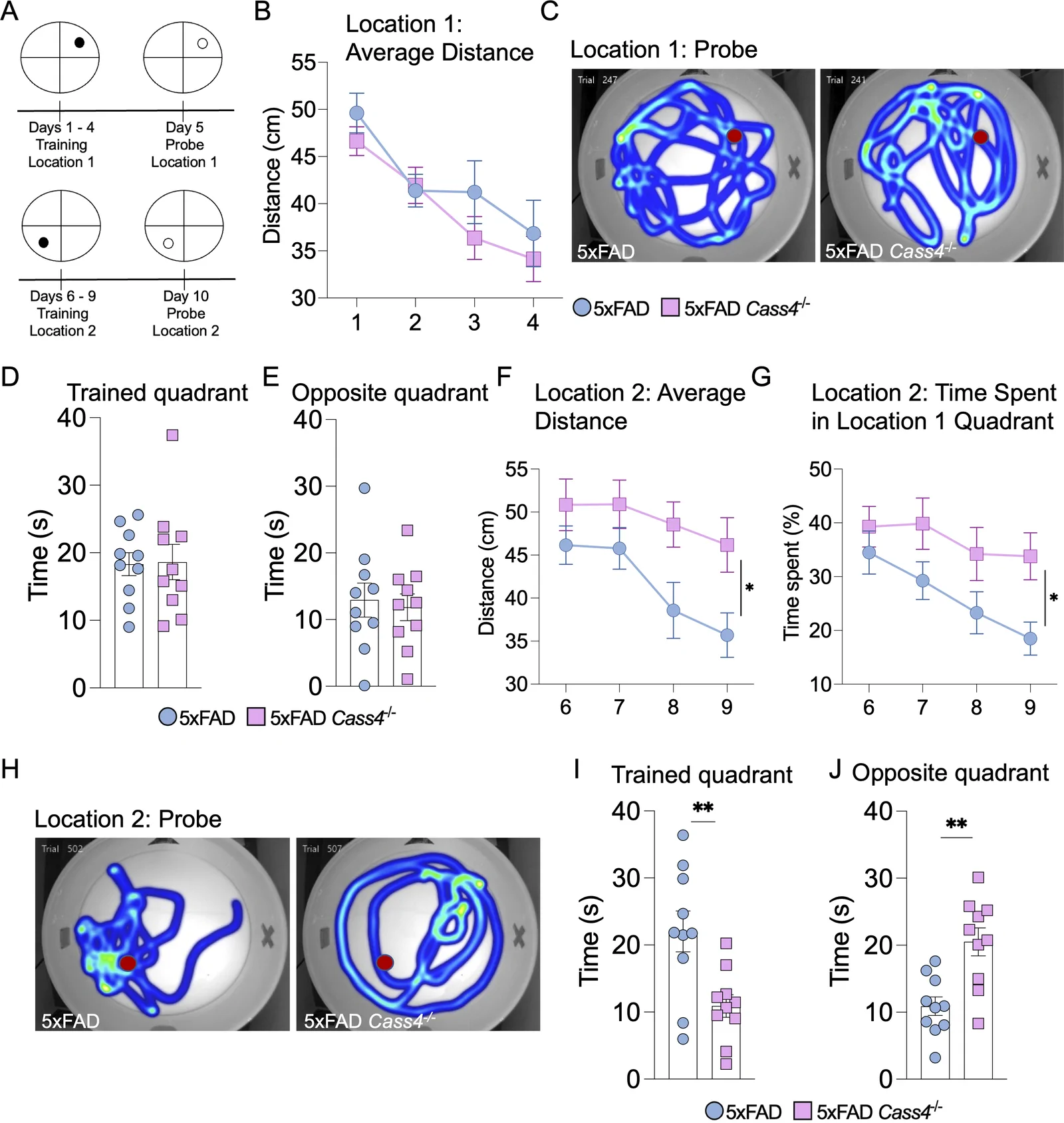
**5xFAD *Cass4*** ^***−/−***^ **mice display impaired cognitive flexibility.** 5xFAD *Cass4*^−/−^ mice and 5xFAD littermate controls were evaluated in the MWM at 4 mo of age. **(A)** Schematic of the MWM testing paradigm performed. **(B)** Average distance mice swam from the platform during the four location 1 training trials. **(C)** Representative heat maps of mouse movement during the location 1 probe. The red circle indicates where the hidden platform was located. **(D)** Time spent in the target quadrant during the location 1 probe. **(E)** Time spent in the opposite quadrant during the location 1 probe. **(F)** Average distance mice swam from the platform during the four location 2 training trials. **(G)** Time spent in the location 1 quadrant during the location 2 training trials. **(H)** Representative heat maps of mouse movement during location 2 probe. **(I)** Time spent in the target quadrant during the location 2 probe. **(J)** Time spent in the opposite quadrant during the location 2 probe. Statistics for MWM learning curves (*n* = 10 mice/group) were calculated with a two-way ANOVA and Šídák’s multiple comparisons test, and data points in the line graphs represent means for each experimental group. Statistics for probes 1 and 2 (*n* = 10 mice/group) were calculated with a Welch’s *t* test, and each data point represents an individual mouse. Combined data from three independent experiments. Error bars represent mean ± SEM. *P < 0.05, **P < 0.01.

Next, we were interested in evaluating how *Cass4* ablation impacts cognitive functions that are disrupted in AD patients. To this end, we evaluated the performance of 4-mo-old 5xFAD and 5xFAD *Cass4*^*−/−*^ mice in the Morris water maze (MWM) (Fig. 3 A), which is a time point when 5xFAD mice have been reported to begin exhibiting cognitive deficits (Ganesh et al., 2024). Interestingly, we found that loss of CASS4 led to pronounced impairments in cognitive flexibility without altering either spatial learning or memory (Fig. 3). More specifically, we saw that both 5xFAD *Cass4*^*−/−*^ mice and 5xFAD littermate controls similarly learned where the platform was over the course of four training days (Fig. 3 B), and we did not uncover any significant differences in the memory probe trial phase of the test (Fig. 3, C–E). In stark contrast, upon moving the platform to a new location (referred to as location 2) and training the mice for an additional 4 days, we observed that 5xFAD *Cass4*^*−/−*^ mice traveled a significantly greater average distance from the new location 2 platform compared with their 5xFAD littermate controls (Fig. 3 F). Additionally, 5xFAD *Cass4*^*−/−*^ mice spent significantly more time swimming in the vicinity of the original target platform (i.e., location 1), as opposed to that of the new location 2 platform (Fig. 3 G). In contrast, 5xFAD littermates spent most of their time in the newly learned location 2 target quadrant and less time in the original location 1 target quadrant, which is indicative of intact cognitive flexibility (Fig. 3 G). We also revealed a similar cognitive flexibility impairment during the location 2 probe trial phase of the test, where 5xFAD *Cass4*^*−/−*^ mice spent significantly less time in the new location 2 target quadrant and significantly more time in the opposite quadrant, which was the location 1 target quadrant (Fig. 3, H–J). In total, these MWM performance findings suggest that 5xFAD *Cass4*^*−/−*^ mice struggled to learn a new task and had deficits in cognitive flexibility.

**Figure S2.**
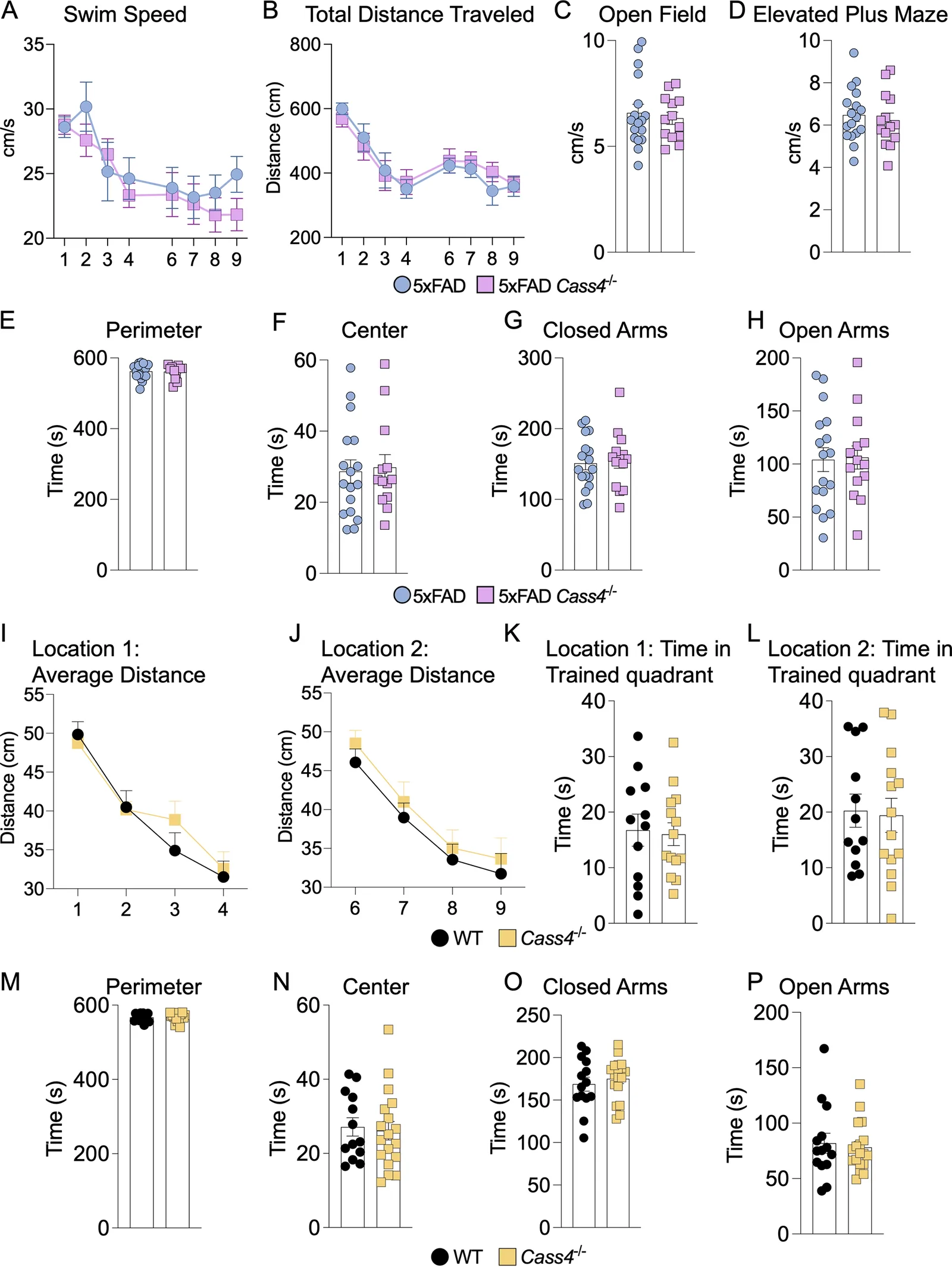
**Effects of *Cass4* deletion on behavior. (A–H)** 5xFAD *Cass4*^−/−^ mice and 5xFAD littermate controls were evaluated in the MWM, open field, and elevated plus maze at 4 mo of age. **(A and B)** Swim speed (A) and total distance traveled (B) by 5xFAD and 5xFAD *Cass4*^−/−^ mice over the location 1 and location 2 training trials of the MWM (*n* = 10 mice/group). **(C and D)** Mouse velocity in the open field test (C) and the elevated plus maze (D). **(E and F)** Time spent in the perimeter (E) and the center of the arena (F) during the open field test. **(G and H)** Time spent in the closed (G) and open (H) arms during the elevated plus maze. **(I–P)***Cass4*^−/−^ mice and WT littermate controls were evaluated in the MWM, open field, and elevated plus maze at 4 mo of age. **(I)** Average distance WT (*n* = 12) and *Cass4*^−/−^ (*n* = 14) mice swam from the platform during the location 1 training trials. **(J)** Average distance mice swam from the platform during the location 2 training trials. **(K)** Time spent in the target quadrant during the location 1 probe. **(L)** Time spent in the target quadrant during the location 2 probe. **(M and N)** Time spent in the perimeter (M) and the center (N) of the arena during the open field test. **(O and P)** Time spent in the closed (O) and open (P) arms during the elevated plus maze. Error bars represent mean ± SEM, and each data point represents an individual mouse. Combined data from three independent experiments. Statistics for MWM velocity and distance curves were calculated with a two-way ANOVA and Šídák’s multiple comparisons test. Data points in the line graphs represent means for each experimental group. Statistical significance between groups for the open field test and elevated plus maze was calculated by unpaired Welch’s *t* test.

Importantly, we did not detect any significant differences in swim speed or total distance traveled between 5xFAD *Cass4*^*−/−*^ and 5xFAD mice over the course of the MWM testing paradigm (Fig. S2, A and B), which indicates that altered locomotor activity likely did not impact performance in these MWM studies. Likewise, we did not observe any appreciable differences in locomotor activity or anxiety-related behavior measurements between 5xFAD *Cass4*^−/−^ mice and 5xFAD littermate controls in the open field or elevated plus maze tests (Fig. S2, C–H). Moreover, *Cass4*-deficient mice that lacked the 5xFAD transgene (i.e., *Cass4*^−/−^ mice) were found to perform comparably with WT littermate controls in the MWM (Fig. S2, I–L), open field (Fig. S2, M and N), and elevated plus maze (Fig. S2, O and P) tests, which suggests that the deficits in cognitive flexibility observed in 5xFAD *Cass4*^−/−^ mice are in response to Aβ pathology. These collective behavioral findings indicate that *Cass4* deletion in 5xFAD mice leads to pronounced deficits in cognitive flexibility, which is notable given that cognitive inflexibility is a common, yet poorly understood, type of memory disruption reported in AD patients (Albert, 1996; Sanchez et al., 2017; Silva-Filho et al., 2007).

### Microglial engagement with Aβ plaques is reduced in the absence of CASS4

**Figure 4.**
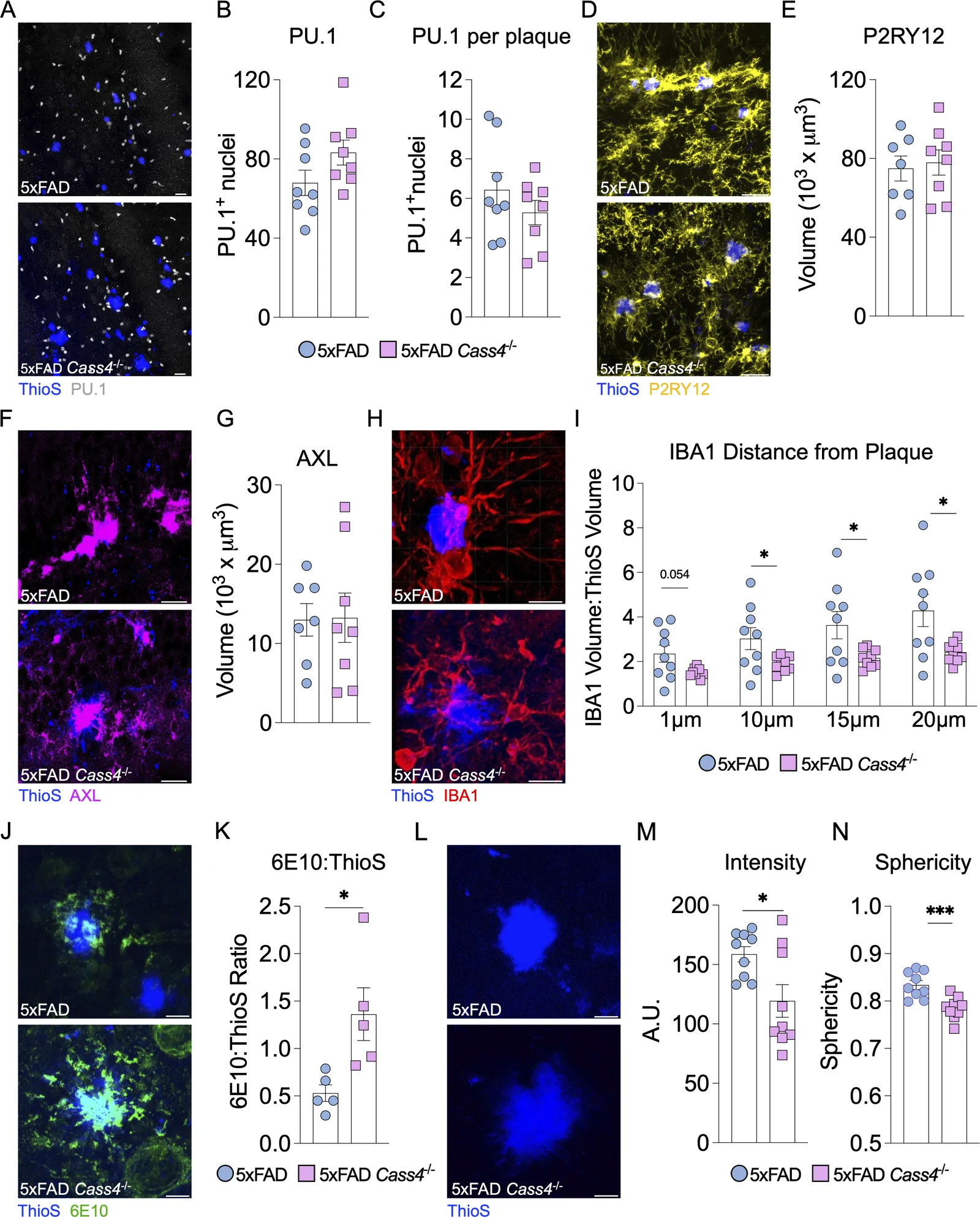
**Loss of CASS4 leads to impaired microglial engagement at Aβ plaques.** Brains from 5xFAD *Cass4*^−/−^ mice and 5xFAD littermate controls were harvested at 5 mo of age to evaluate microglial characteristics by confocal imaging. **(A)** Representative images of Aβ plaque (ThioS, blue) and microglial nuclei (PU.1, gray) in the hippocampus at 40× magnification. Scale bar: 20 μm. **(B)** PU.1^+^ microglial nuclei count per FOV in the hippocampus. **(C)** PU.1^+^ microglia per ThioS^+^ Aβ plaque per FOV in the hippocampus. **(D)** Representative images of Aβ plaque (ThioS, blue) and P2RY12 (yellow) in the hippocampus at 40× magnification. Scale bar: 20 μm. **(E)** The total volume of P2RY12 overlapped with IBA1 in the hippocampus per FOV. **(F)** Representative images of Aβ plaque (ThioS, blue) and AXL (pink) in the hippocampus at 40× magnification. Scale bar: 20 μm. **(G)** Total volume of AXL overlapped with IBA1 in the hippocampus per FOV. **(H)** Representative images of Aβ plaque (ThioS, blue) and microglia processes (IBA1, red) in the hippocampus at 63× magnification. Scale bar: 10 μm. **(I)** IBA1 volume-to-ThioS volume ratio within 1, 10, 15, and 20 μm of ThioS^+^ Aβ plaque in the hippocampus. **(J)** Representative images of dense core Aβ plaque (ThioS, blue) and diffuse Aβ (6E10, green) in the hippocampus at 40× magnification. Scale bar: 50 μm. **(K)** 6E10 to ThioS ratio in the hippocampus. **(L)** Representative images of Aβ plaque (ThioS, blue) sphericity and intensity in the hippocampus at 63× magnification. Scale bar: 20 μm. **(M)** Quantification of plaque intensity. **(N)** Quantification of plaque sphericity (a value of 1 is a perfect sphere). For all imaging experiments, three brain sections per mouse were imaged, with at least three FOV taken per section per region (a total of 9–12 images per mouse). Statistical significance between experimental groups was calculated by a linear mixed-effects model. *P < 0.05, ***P < 0.001. Error bars represent the mean ± SEM, and each data point represents an individual mouse.

**Figure S3.**
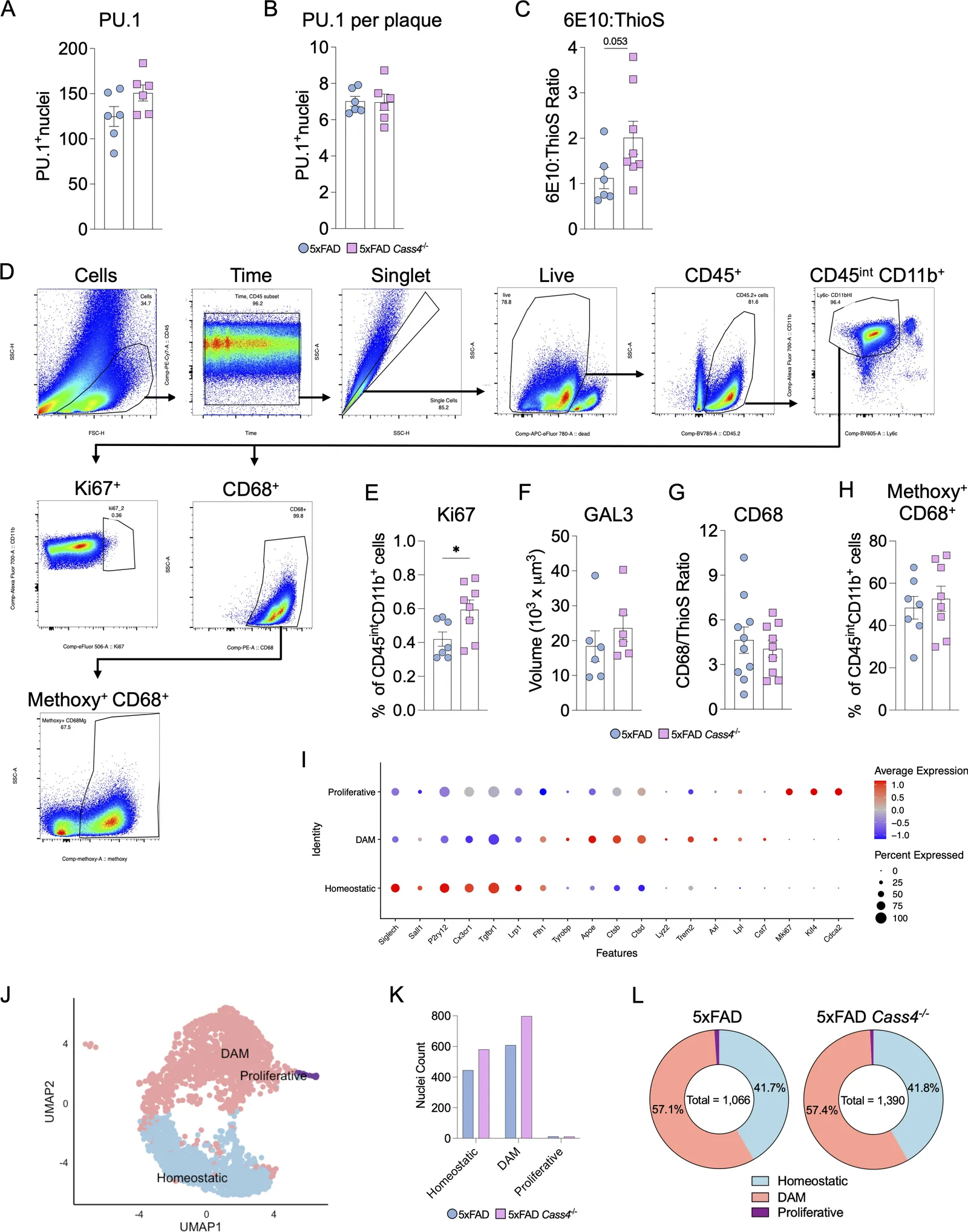
**Effect of *Cass4* deletion on Aβ pathology and microglial responses in 5xFAD mice. (A–C)** 5xFAD *Cass4*^−/−^ mice and 5xFAD littermate controls were harvested at 5 mo of age to evaluate Aβ load and microglial nuclei by confocal imaging. **(A)** PU.1^+^ microglial nuclei counts per FOV in the cortex taken at 40×. **(B)** PU.1^+^ microglial nuclei per ThioS^+^ Aβ plaque per FOV in the cortex. **(C)** 6E10 to ThioS ratio of the cortex taken at 40×. **(D, E, and H)** Brains from 5xFAD *Cass4*^−/−^ mice and 5xFAD littermate controls were harvested at 5 mo of age to evaluate microglial characteristics by confocal imaging or flow cytometry. **(D)** Gating strategy to characterize microglia. Cells were selected from a forward scatter-height versus side scatter-height dot plot, followed by a control time gate (PE-Cy7 versus time). Single cells were subsequently selected in a side scatter-area versus side scatter-height dot plot, and live cells were selected as negative for Live/Dead stain. Leukocytes were selected as CD45^+^. The microglia were gated from the CD45^+^ gate (CD11b^+^Ly6C^neg^CD45^int^). Proliferative and phagocytic subsets were gated from the microglia as Ki67^+^ and CD68^+^, respectively. Lastly, the Aβ phagocytic population was determined as methoxy^+^. **(E)** The proportion of Ki67^+^ cells within the CD45^i^^nt^CD11b^hi^ flow cytometry gate. **(F)** The total volume of GAL3 overlapped with IBA1 in the hippocampus per FOV taken at 40×. **(G)** The total volume of CD68 overlapped with IBA1 and then normalized to total ThioS volume in the hippocampus per FOV taken at 63×. **(H)** 5-mo-old 5xFAD *Cass4*^−/−^ mice and 5xFAD littermate controls received intraperitoneal injections of methoxy-X04, and then brains were harvested 3 h later to evaluate microglial phagocytosis of methoxy-X04^+^–labeled Aβ via flow cytometry. The proportion of CD68^+^ methoxy-X04^+^ cells within the CD45^i^^nt^CD11b^hi^ flow cytometry gate was then determined. **(I–L)** snRNA-seq was performed on 5xFAD *Cass4*^−/−^ mice and 5xFAD littermate controls (*n* = 6 mice/group). The microglia were further subclustered. **(I)** Dot plot depicting the nuclei identity of each subcluster, as determined by the expression of cell type-specific markers. **(J)** UMAP plot depicting three unique microglia subclusters. **(K)** Bar chart showing the nuclei distribution per cell type in each group. **(L)** Donut chart depicting the frequency of nuclei recovered per cell type across both groups. For all imaging experiments, three brain sections per mouse were imaged, with at least three FOV taken per section per region (a total of 9–12 images per mouse). For imaging experiments (*n* = 6 mice/group), statistical significance between experimental groups was calculated by a linear mixed-effects model. For flow cytometry experiments (*n* = 6–7 mice/group), statistical significance between groups was calculated by unpaired Welch’s *t* test. Error bars represent mean ± SEM, and each data point represents an individual mouse. *P < 0.05.

To better understand what contributes to exacerbated amyloidosis and neuronal dystrophy in CASS4-deficient 5xFAD mice, we next turned our attention to microglia, given the ever-increasing evidence linking defective microglial biology to AD pathogenesis, as well as the fact that microglia are the sole brain-resident cell type that expresses *Cass4* (Fig. S1 G). We first investigated whether microglial numbers were altered in 5xFAD *Cass4*^*−/−*^ mice, as microglia are known to migrate and proliferate in the presence of Aβ plaques (Bolmont et al., 2008; Nimmerjahn et al., 2005; Olmedillas et al., 2023). Despite the increase in Aβ pathology seen in CASS4-deficient 5xFAD mice (Fig. 1), we found that the numbers of nuclei in the hippocampus and cortex expressing the macrophage marker PU.1 were similar between 5xFAD and 5xFAD *Cass4*^*−/−*^ mice (Fig. 4, A and B; and Fig. S3 A). Furthermore, we also did not observe any appreciable differences in PU.1^+^ nuclei when normalized to Aβ plaque numbers between 5xFAD *Cass4*^*−/−*^ mice and 5xFAD littermate controls (Fig. 4 C and Fig. S3 B). As expected, with the lack of differences in hippocampal or cortical PU.1^+^ microglia numbers (Fig. 4, A–C), we also found that *Cass4* deletion in 5-mo-old 5xFAD mice did not appreciably impact the proportion of proliferative Ki67^+^CD45^int^ CD11b^+^ microglia in flow cytometry studies (Fig. S3, D and E).

**Figure S4.**
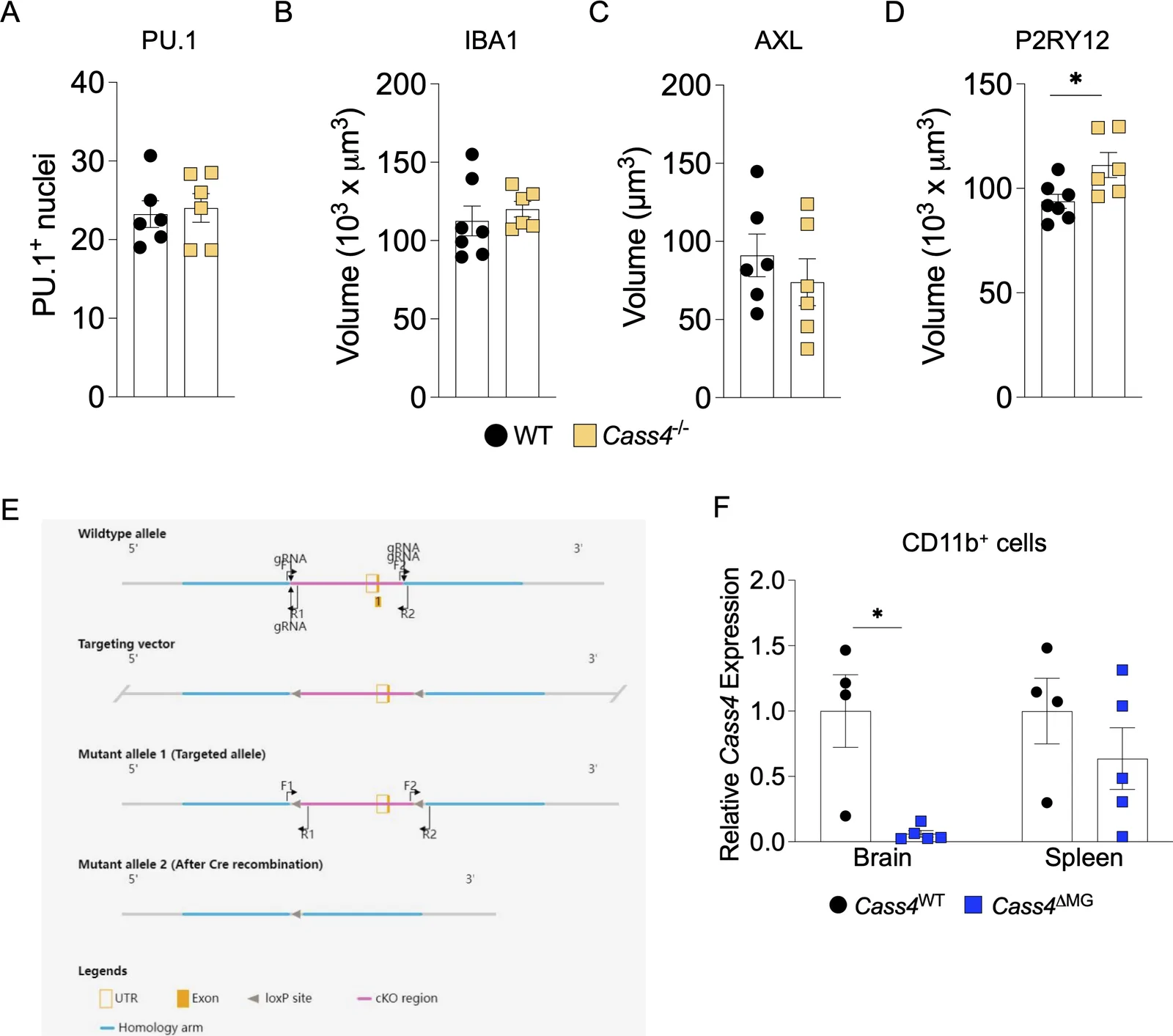
**Impact of *Cass4* deletion on microglial molecular marker expression in the absence of amyloid pathology and generation of *Cass4*** ^**fl/fl**^ ***Cx3cr1*** ^**ERT2Cre**^ **mice. (A–D)** Brains from *Cass4*^−/−^ mice and WT littermate controls were harvested at 5 mo of age to evaluate microglial responses in the absence of amyloid pathology by confocal imaging. **(A)** PU.1^+^ microglia nuclei per FOV in the hippocampus in WT and *Cass4*^−/−^ mice. **(B)** Quantification of IBA1 volume per FOV in the hippocampus. **(C)** Quantification of AXL overlapped with IBA1 volume per FOV in the hippocampus. **(D)** Quantification of P2RY12 overlapped with IBA1 volume per FOV in the hippocampus. **(E)** Gene targeting strategy to generate *Cass4*^fl/fl^ mice. **(F)***Cass4*^fl/fl^*Cx3cr1*^ERT2Cre^ mice (*Cass4*^ΔMG^ mice) and Cre-negative *Cass4*^fl/fl^ littermate controls (*Cass4*^WT^ mice) received tamoxifen chow at 3 wk of age for 2 wk and then were returned to regular chow for the remainder of the experiment. Mice were harvested at 5 mo of age, and CD11b^+^ macrophages were isolated from the spleen and brain by magnetic bead–based purification. Relative expression of *Cass4* was then determined by qPCR. For all imaging experiments, three brain sections per mouse were imaged at 40× magnification, with at least three FOV taken per section per region (a total of 9–12 images per mouse). Statistical significance between experimental groups was calculated by a linear mixed-effects model. Statistics for qPCR were calculated with a Welch’s *t* test, and each data point represents an individual mouse. Error bars represent mean ± SEM. *P < 0.05.

The activation status of microglia recruited to Aβ pathology also has a significant bearing on their ability to effectively control amyloidosis in AD. More specifically, controlled activation of microglia and acquisition of the disease-associated microglia (DAM) phenotype (also referred to as the microglia neurodegenerative phenotype) is known to equip microglia with neuroprotective properties needed to optimally protect against Aβ amyloidosis (Keren-Shaul et al., 2017; Krasemann et al., 2017). However, to our surprise, we did not detect any major differences in the expression of the homeostatic microglial marker P2RY12 or the activation markers AXL, GAL3, or CD68 between hippocampal IBA1^+^ microglia in 5-mo-old *Cass4*-deficient 5xFAD versus 5xFAD littermate controls (Fig. 4, D–G; and Fig. S3, F and G). In agreement with these imaging results (Fig. 4, D–G), the proportions of homeostatic microglia and DAM were also found to be equivalent between 5xFAD *Cass4*^−/−^ and 5xFAD mice in our snRNA-seq analysis (Fig. S3, I–L). Similarly, loss of *Cass4* was not found to affect the number of microglia, microgliosis, or the expression of the DAM marker AXL by IBA1^+^ cells (Fig. S4, A–C) in the absence of amyloid pathology. However, we did detect slightly elevated staining of the homeostatic marker P2RY12 in *Cass4*^−/−^ mice relative to WT controls (Fig. S4 D).

Phagocytosis is another strategy deployed by microglia to restrain Aβ pathology in AD. To investigate if *Cass4* deletion impacts phagocytosis, 5-mo-old 5xFAD and 5xFAD *Cass4*^*−/−*^ mice received an intraperitoneal injection of methoxy-X04, which is a brain-penetrant dye that labels fibrillar Aβ. Brains were then harvested 3 h later, and the percentage of brain CD45^int^ CD11b^+^ microglia that took up methoxy-X04–labeled Aβ was determined by flow cytometry (Fig. S3 D). Similar to our microglial numbers and activation status findings, we did not observe appreciable differences in the uptake of methoxy-X04^+^ Aβ species by CD45^int^ CD11b^+^ microglia from 5-mo-old 5xFAD and 5xFAD *Cass4*^*−/−*^ mice (Fig. S3 H). In total, these findings suggest that genetic ablation of *Cass4* does not impact microglial numbers, DAM phenotype, or phagocytic activity in response to Aβ pathology.

As another neuroprotective strategy, microglia also migrate to and encapsulate Aβ plaque surfaces to limit toxicity (Condello et al., 2015; Wang et al., 2016). When Aβ species are diffuse, there is more surface area available to interfere with nearby neurons, and thus the toxic effects on the brain parenchyma are increased compared with compacted dense core Aβ plaques (Ennerfelt et al., 2022; Yuan et al., 2016). Since we did not see any appreciable differences in the number of PU.1^+^ microglia nuclei in relation to Aβ plaques (Fig. 4, A–C; and Fig. S3, A and B), we next wondered if *Cass4* deficiency leads to impaired containment of Aβ plaques by microglia. To this end, we evaluated IBA1 staining in the area directly adjacent to ThioS^+^ Aβ plaques. Here, we observed markedly less IBA1 volume in close proximity to Aβ plaques in 5xFAD *Cass4*^*−/−*^ mice relative to 5xFAD littermate controls (Fig. 4, H and I). These collective results suggest that CASS4 does not appreciably influence microglia numbers or activation in amyloidosis but instead plays a key role in regulating close interactions of microglia at plaque surfaces.

There are several species of Aβ that can be present in the brain during AD, with soluble Aβ thought to be the most toxic species due to its increased hydrophobicity and diffuse nature as compared with dense core plaques (Casali et al., 2020; Spangenberg et al., 2019). Importantly, microglia have been identified to play especially pivotal roles in limiting the spread of pathological Aβ in the brain by actively compacting diffuse Aβ fibrils into dense spherical plaques in an effort to minimize the surface area of Aβ that can interact with susceptible neurons (Condello et al., 2015; Zhao et al., 2017). Leveraging the 6E10 antibody to stain diffuse Aβ and ThioS as a marker of dense core plaques, we found that 5xFAD *Cass4*^*−/−*^ mice had an increased ratio of 6E10:ThioS in the hippocampus and cortex relative to 5xFAD littermate controls (Fig. 4, J and K; and Fig. S3 C). Assessments of Aβ plaque sphericity and intensity are also often employed to identify potentially neurotoxic Aβ aggregates and to provide insights into the efficacy of microglial compaction. Consistent with our findings demonstrating increased abundance of diffuse 6E10 staining in *Cass4*-deficient 5xFAD mice (Fig. 4, J and K; and Fig. S3 C), we also observed lower intensity and sphericity of Aβ plaques in the hippocampus of 5xFAD *Cass4*^*−/−*^ mice when compared with 5xFAD littermate controls (Fig. 4, L–N). Together, these findings indicate that Aβ plaque compaction is impaired with the loss of CASS4 and further show that this is associated with defects in the ability of microglia to form close contacts with Aβ aggregates.

### Conditional deletion of *Cass4* in microglia results in impaired control of Aβ amyloidosis and exacerbated neuritic dystrophy

**Figure 5.**
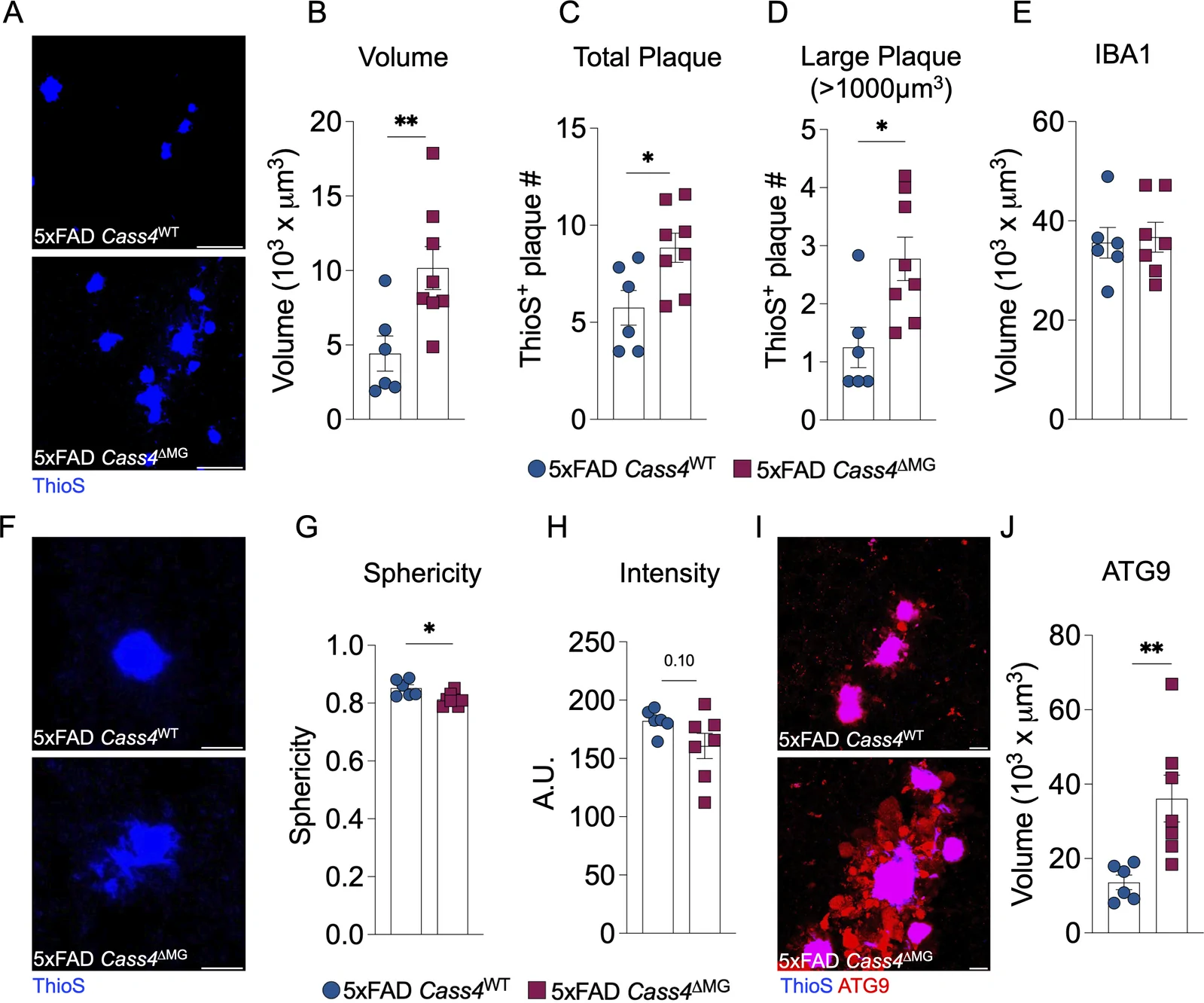
**Microglia-targeted deletion of *Cass4* in 5xFAD mice leads to increased Aβ load and worsened neuronal health.** 5xFAD *Cass4*^fl/fl^*Cx3cr1*^ERT2Cre^ mice (5xFAD *Cass4*^ΔMG^ mice) and Cre-negative 5xFAD *Cass4*^fl/fl^ littermate controls (5xFAD *Cass4*^WT^ mice) received tamoxifen chow at 3 wk of age for 2 wk and then were returned to regular chow for the remainder of the experiment. Mice were harvested at 5 mo of age to evaluate amyloid plaque load, microglial responses, and neuritic dystrophy by confocal imaging. **(A)** Representative images of Aβ plaque (ThioS, blue) in the hippocampus at 63× magnification. Scale bar: 40 μm. **(B)** Total ThioS^+^ Aβ plaque volume per FOV in the hippocampus. **(C)** ThioS^+^ Aβ plaque count per FOV in the hippocampus. **(D)** Large plaque count, as determined by a ThioS volume of >1,000 μm^3^ per Aβ plaque in the hippocampus. **(E)** Total IBA1 volume per FOV in the hippocampus. **(F)** Representative images of Aβ plaque (ThioS, blue) sphericity and intensity in the hippocampus at 63× magnification. Scale bar: 10 μm. **(G)** Quantification of plaque sphericity (a value of 1 is a perfect sphere). **(H)** Quantification of plaque intensity. **(I)** Representative images for dystrophic neurites (ATG9, red) and Aβ plaques (ThioS, blue) in the hippocampus at 63× magnification. Scale bar: 7 μm. **(J)** Total volume of ATG9^+^ puncta in the hippocampus per FOV. For all imaging experiments, three brain sections per mouse were imaged, with at least three FOV taken per section per region (a total of 9–12 images per mouse). Statistical significance between experimental groups was calculated by a linear mixed-effects model. *P < 0.05, **P < 0.01. Error bars represent mean ± SEM, and each data point represents an individual mouse.

To further interrogate a microglial-specific role for CASS4 in amyloidosis, we next generated *Cass4*^fl/fl^*Cx3cr1*^ERT2Cre^ mice to target deletion of *Cass4* from microglia (Fig. S4, E and F) and then crossed them to 5xFAD mice (further referred to as 5xFAD *Cass4*^ΔMG^). 5xFAD *Cass4*^ΔMG^ mice and Cre-negative 5xFAD *Cass4*^fl/fl^ littermate controls (hereafter referred to as 5xFAD *Cass4*^WT^ mice) received tamoxifen food at weaning for 2 wk to induce *Cass4* deletion. Mice were then returned to normal chow to allow for peripheral *Cx3cr1*-expressing cells to turn over and regain *Cass4* expression, while the long-lived microglia maintained *Cass4* deficiency (Yona et al., 2013). Consistent with our amyloidosis findings in germline *Cass4*-deficient mice (Fig. 1), we likewise found that 5-mo-old 5xFAD *Cass4*^ΔMG^ mice had a greater total volume of ThioS^+^ staining and increased numbers of ThioS^+^ Aβ plaques in the hippocampus relative to 5xFAD *Cass4*^WT^ littermate controls (Fig. 5, A–C). 5xFAD *Cass4*^ΔMG^ mice also had elevated numbers of large plaques in the hippocampus, which were defined as plaques >1,000 μm^3^ in volume (Fig. 5 D). While 5xFAD *Cass4*^ΔMG^ mice also showed no differences in overall IBA1 volume (Fig. 5 E), we observed a similar reduction in plaque sphericity and intensity to that observed in germline *Cass4*-deficient mice (Fig. 4, L–M) in 5xFAD *Cass4*^ΔMG^ mice when compared with 5xFAD *Cass4*^WT^ controls (Fig. 5, F–H). Lastly, similar to our *Cass4* germline knockout findings that loss of *Cass4* worsens neuronal dystrophy in 5xFAD mice (Fig. 2, C–E), we also found that microglial-targeted deletion of *Cass4* leads to increased levels of hippocampal ATG9 staining in 5xFAD *Cass4*^ΔMG^ mice (Fig. 5, I and J), which is indicative of worsened neuronal health. These data suggest that microglia-targeted deletion of *Cass4* recapitulates the amyloidosis and neuritic dystrophy phenotypes seen in full-body CASS4-deficient 5xFAD mice (Figs. 1, 2, and 4) and further indicate that CASS4 functions in CNS-resident macrophages to impact amyloidosis.

### CASS4 modulates focal adhesion-associated processes and signaling in microglia

**Figure 6.**
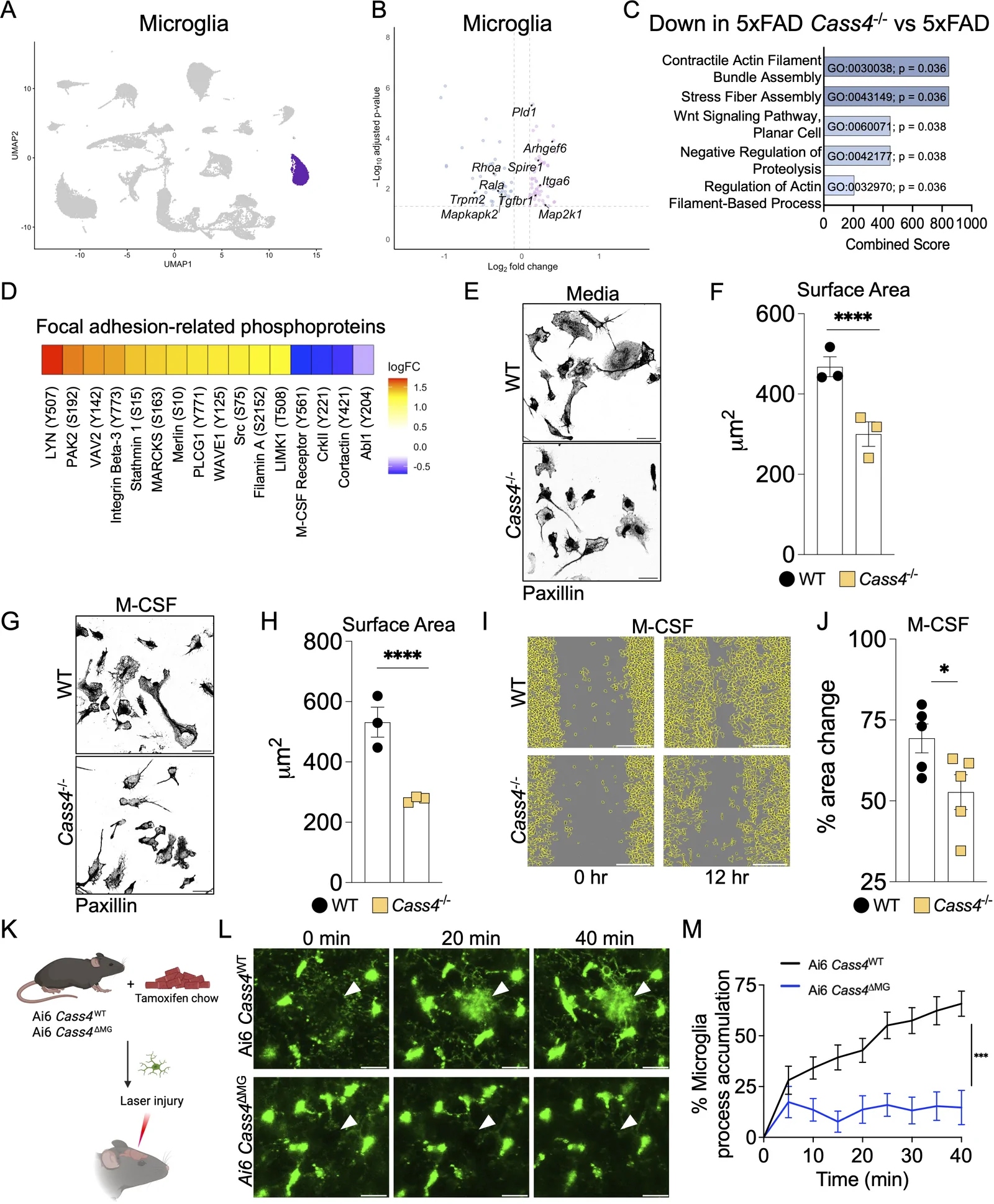
**CASS4 regulates focal adhesion-related processes and signaling in microglia.** Hippocampi from 5xFAD *Cass4*^−/−^ mice and 5xFAD littermate controls were microdissected at 5 mo of age and pooled (*n* = 6 mice/group) for snRNA-seq. **(A)** UMAP plot displaying the microglia cluster. **(B)** Volcano plot depicting DEGs (upregulated, pink; downregulated, blue) in the microglia cluster of 5xFAD *Cass4*^−/−^ versus 5xFAD microglia. Colored points depict genes with adjusted P values <0.05 and log_2_ fold changes ±0.1. **(C)** Bar plots depicting downregulated GO terms enriched in 5xFAD *Cass4*^*−/−*^ microglia. Functional enrichment analysis was performed with g:Profiler using genes with adjusted P values <0.05 and log_2_ fold changes ±0.25. Depicted pathways were curated from the top driver terms in GO. **(D)** Phosphoprotein analysis was performed on CD11b^+^ microglia isolated by magnetic bead sorting from 5xFAD *Cass4*^*−/−*^ mice and 5xFAD littermate controls at 5 mo of age (*n* = 5 mice/group). Select differentially enriched phosphoproteins (log fold change >1.4 or < −0.4). **(E)** Representative images of primary microglia plated onto laminin-coated coverslips in the presence of media alone. Scale bar: 20 μm. **(F)** Surface area of paxillin staining in the presence of media alone. **(G)** Representative images of primary microglia plated on laminin-coated coverslips in the presence of media containing 50 ng/ml M-CSF. Scale bar: 20 μm. **(H)** Surface area of paxillin from primary microglia in the presence of media containing 50 ng/ml M-CSF. Paxillin data are pooled from three independent experiments and 12 FOVs imaged per experiment (*n* = 3 per group). **(I)** Representative images of WT and *Cass4*^*−/−*^ BMDMs in media containing 50 ng/ml M-CSF before and 12 h following scratch wound creation. Scale bar: 400 μm. **(J)** Quantification of percent change in wound area over 12 h. Scratch assay data are pooled from three independent experiments and are three technical replicates per mouse (*n* = 5 per group). Statistics for scratch assay percent area change were calculated with a Welch’s *t* test. **(K)** Schematic depicting the use of Ai6^+/−^*Cass4*^+/+^*Cx3cr1*^ERT2Cre^ (Ai6 *Cass4*^WT^) and Ai6^+/−^*Cass4*^fl/fl^*Cx3cr1*^ERT2Cre^ (Ai6 *Cass4*^ΔMG^) mice for in vivo imaging after a laser injury. **(L)** Representative in vivo two-photon images from a time-lapse collected on Ai6^+/−^ microglia (green) in the cortex with or without *Cass4* expression following a laser-induced injury (white arrow denotes the site of laser injury). Scale bar: 20 μm. **(M)** Quantification of percent microglial process change around the laser injury target area as assessed by changes in Ai6^+^ microglial process area over time. Two-photon imaging was performed on four to five mice per group with one to two FOV per animal, and data were combined from three independent experiments. Percent area change and percent microglial process change statistics were calculated by two-way ANOVA and Šídák’s multiple comparisons test. Data points in the line graph represent means for each experimental group. Error bars represent mean ± SEM. *P < 0.05, ***P < 0.001, and ****P < 0.0001. GO, Gene Ontology.

To gain additional insights into how CASS4 shapes microglial biology in AD-related amyloidosis, we returned to our snRNA-seq dataset comparing hippocampal gene expression between 5-mo-old 5xFAD *Cass4*^−/−^ and 5xFAD mice (Fig. S1, C–G). From the whole-brain snRNA-seq dataset (Fig. S1, C–G), we extracted the microglia cluster and performed differential gene expression analysis (Fig. 6, A and B) and microglial subset re-clustering (Fig. S3, I–L). For the full microglia cluster, we identified 98 DEGs between 5xFAD and 5xFAD *Cass4*^−/−^ microglia (Fig. 6 B). Pathway analysis of these DEGs identified that multiple processes associated with focal adhesion biology were downregulated in 5xFAD *Cass4*^−/−^ microglia relative to 5xFAD microglia (Fig. 6 C). Most notably, analysis in the microglia cluster revealed that loss of CASS4 in 5xFAD mice was associated with a downregulation of genes linked to contractile actin filament assembly, stress fiber assembly, and regulation of actin filament-based processes (Fig. 6 C; Ahn and Park, 2025; Biermeier et al., 2026; Deveci et al., 2026; Li et al., 2016; Malko et al., 2019; Nobes and Hall, 1995; Ridley, 2001; Zhang et al., 2019), all of which are molecular processes centrally involved in focal adhesion biology (Burridge and Guilluy, 2016; Ciobanasu et al., 2012; Geiger et al., 2009; Wozniak et al., 2004).

**Figure S5.**
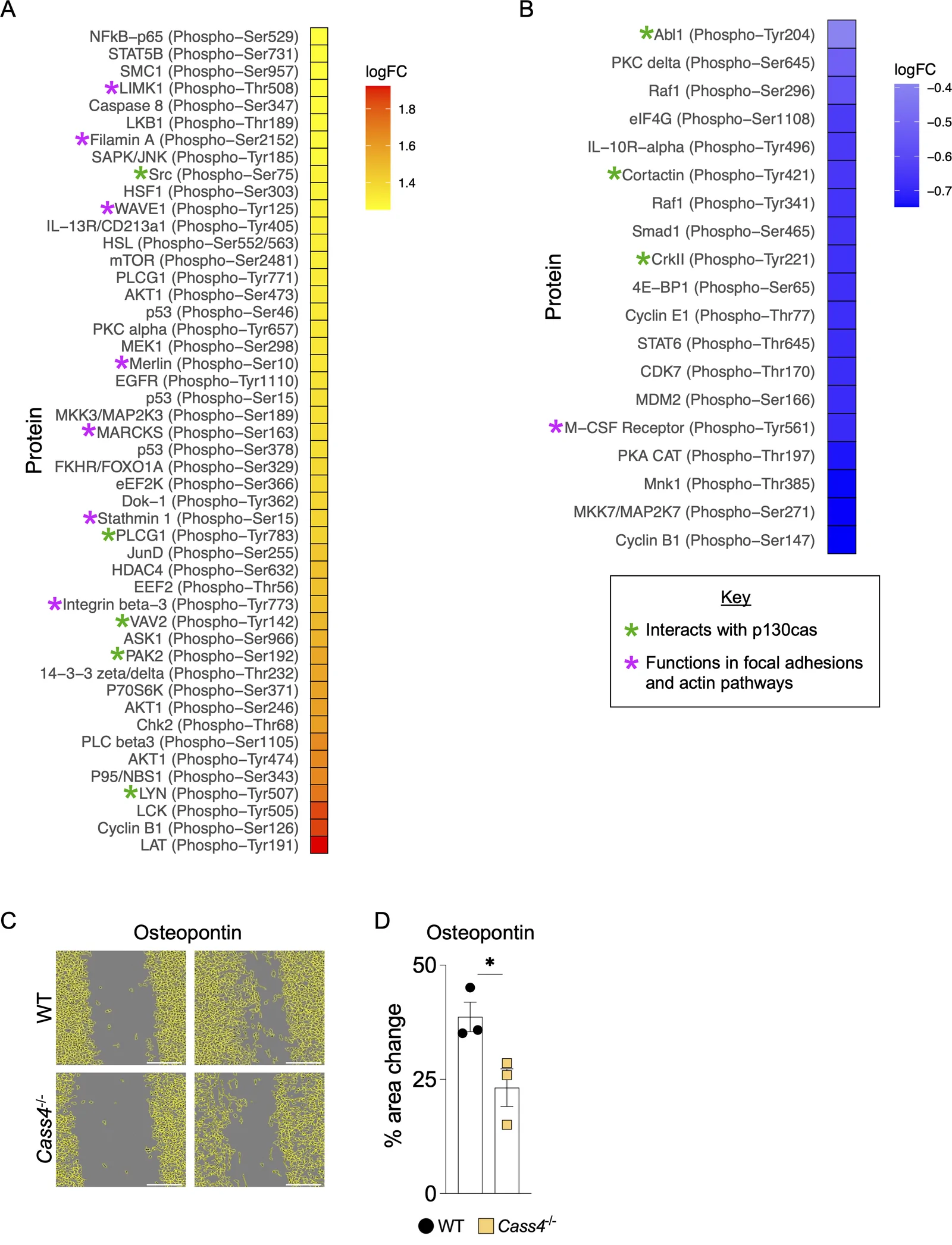
**Genetic ablation of *Cass4* in 5xFAD mice leads to altered focal adhesion signaling in microglia and impaired macrophage recovery following scratch injury. (A and B)** Phosphoprotein analysis was performed on CD11b^+^ cells that were isolated by magnetic bead–based purification from 5xFAD *Cass4*^*−/−*^ mice and 5xFAD littermate controls at 5 mo of age (*n* = 5 mice/group). **(A)** All differentially upregulated phosphoproteins (log fold change >1.4) in 5xFAD *Cass4*^*−/−*^ microglia. **(B)** All differentially downregulated phosphoproteins (log fold change <−0.4) in 5xFAD *Cass4*^*−/−*^ microglia. Data averaged across two independent experiments. Green asterisks denote proteins that are known to interact with the focal adhesion protein p130cas and pink asterisks denote proteins that function in focal adhesion and actin pathways. **(C)** Representative images of WT and *Cass4*^*−/−*^ BMDMs in media containing 400 ng/ml osteopontin before and 12 h following scratch wound creation. Scale bar: 400 μm. **(D)** Quantification of percent change of the wound area over 12 h. Scratch assay data are pooled from three independent experiments and are three technical replicates per mouse (*n* = 5 mice per group). Statistics for scratch assay percent area change were calculated with a Welch’s *t* test. *P < 0.05. Error bars represent mean ± SEM, and each data point represents an individual mouse.

Given that focal adhesion biology is known to be regulated predominantly at the posttranslational level, we next turned to a proteomics-based approach to further interrogate whether CASS4 is involved in the regulation of focal adhesion signaling. To this end, we isolated microglia from the brains of 5-mo-old 5xFAD and 5xFAD *Cass4*^*−/−*^ mice by performing magnetic-activated cell sorting (MACS) for CD11b^+^ cells. We then performed broad-scope protein phosphorylation profiling by running protein lysates from the isolated microglia on an antibody array with 1,318 site-specific and phospho-specific antibodies from over 30 signaling pathways. Here, we observed that of the 93 proteins identified to exhibit differential phosphorylation status between 5xFAD and 5xFAD *Cass4*^*−/−*^ microglia, 17% are known to be central molecular players in focal adhesion signaling (Fig. 6 D and Fig. S5, A and B; Farhadi and Park, 2025; Fujita et al., 2016; Geiger, 2006; Henderson et al., 2019; Huveneers and Danen, 2009; Islam et al., 2025; Ma et al., 2008; Nakamura et al., 2001; Zhang et al., 2022). This included decreased phosphorylation of CrkII, cortactin, and Abl1 in 5xFAD *Cass4*^*−/−*^ microglia relative to microglia isolated from 5xFAD littermate controls (Fig. 6 D). Of note, CrkII, cortactin, and Abl1 are each key downstream targets of focal adhesion signaling, where they contribute to cell migration, actin-based cytoskeleton dynamics, and the formation of cellular protrusions such as filopodia and lamellipodia (Duong et al., 1998; Gustavsson et al., 2004; Jin and Wang, 2007; Lamorte et al., 2003; Lee et al., 2007; Tehrani et al., 2006; Zhou et al., 2020). Moreover, we also noted that 5xFAD *Cass4*^*−/−*^ microglia displayed increased phosphorylation of multiple apical focal adhesion-related proteins such as integrin β3, Src, filamin A, and LYN (Fig. 6 D; Bellenguez et al., 2022; Feng and Walsh, 2004; Gao et al., 2021; Kim et al., 2010; Ortiz et al., 2021; Pfaff and Jurdic, 2001; Portugal et al., 2022), which could be upregulated in an effort to compensate for the defects further downstream in this pathway (i.e., CrkII, cortactin, and Abl1). Interestingly, focal adhesion signaling has recently been linked to AD through both genetic risk factors and Aβ-associated signaling events (Grace and Busciglio, 2003; Lambert et al., 2013; Lin et al., 2017; Manocha et al., 2015; Ramos de Matos et al., 2018; Saleh et al., 2022; Schwartzentruber et al., 2021; Wang et al., 2015b), yet the contribution of this pathway to microglial biology and AD is currently poorly understood.

Focal adhesions are positioned at critical junctions of cells—linking the extracellular matrix with the actin cytoskeleton—and allowing microglia to efficiently survey the parenchyma and respond to injuries (Choi et al., 2015; Meller et al., 2017). Focal adhesions are also known to play critical roles in both M-CSF- and osteopontin-mediated migration and wound resolution in scratch assays (Dashdulam et al., 2020; Murrey et al., 2020). Interestingly, one of the proteins that had reduced phosphorylation in 5xFAD *Cass4*^*−/−*^ microglia was the M-CSF receptor (P-Y561) (Fig. 6 D). M-CSF signaling is crucial to brain macrophage function, and phosphorylation of Y561 is known to relieve autoinhibition of the M-CSF receptor (Stanley and Chitu, 2014). Our observation of reduced P-Y561 suggests that *Cass4*^−/−^ microglia may have reduced signaling capacity through the M-CSF pathway and consequently have dampened migratory responses. Consistent with a role for CASS4 in focal adhesion biology, we observed that *Cass4-*deficient primary microglia exhibited reduced surface area of the focal adhesion protein paxillin in response to laminin-coated coverslips (Fig. 6, E and F). This reduced surface area was also detected upon treatment with 50 ng/ml M-CSF (Fig. 6, G and H), suggesting that loss of CASS4 reduces microglial responses to M-CSF. To provide a proof-of-concept understanding of cellular migration upon loss of *Cass4*, we used *Cass4*-deficient bone marrow–derived macrophages (BMDMs) exposed to a standard scratch assay experimental paradigm, which allowed us to assess the collective migration of cells in response to a physical scratch injury (Grada et al., 2017; Jonkman et al., 2014). In line with our previous observations, *Cass4-*deficient BMDMs exhibited reduced migration in response to M-CSF (Fig. 6, I and J). Additionally, *Cass4*-deficient BMDMs also displayed diminished migration in scratch assays when treated with 400 ng/ml osteopontin (Fig. S5, C and D), another molecule known to leverage focal adhesion signaling to influence cellular responses and wound healing (Azizan et al., 2025; Dashdulam et al., 2020).

To evaluate if CASS4 is also involved in coordinating in vivo microglial responses to injury, we next leveraged two-photon imaging and Ai6 reporter mice that were crossed to *Cass4*^fl/fl^*Cx3cr1*^ERT2Cre^ mice to track microglial dynamics following laser injury (Merlini et al., 2021; Nimmerjahn et al., 2005; Tognatta et al., 2021). To this end, Ai6^+/−^*Cass4*^+/+^*Cx3cr1*^ERT2Cre^ (Ai6 *Cass4*^WT^) and Ai6^+/−^*Cass4*^fl/fl^*Cx3cr1*^ERT2Cre^ (Ai6 *Cass4*^ΔMG^) mice received tamoxifen food at weaning for 2 wk to induce *Cass4* deletion and Ai6 expression to track in vivo microglial dynamics (Fig. 6 K). Mice were then returned to normal chow for 1 mo to allow for peripheral *Cx3cr1*-expressing cells to turn over and regain *Cass4* expression, while allowing long-lived microglia to maintain Ai6 fluorescence and *Cass4* deficiency (Yona et al., 2013). We observed that microglial processes vigorously converged to the site of laser injury in Ai6^+/−^*Cass4*^WT^ mice (Fig. 6, L and M; and Video 1). In stark contrast, microglia lacking CASS4 displayed blunted mobilization to the laser injury (Fig. 6, L and M; and Video 2). Altogether, these results indicate that the absence of CASS4 in microglia alters focal adhesion signaling and results in impairments to cellular processes that are known to be regulated by focal adhesions, such as microglia and macrophage mobilization to injury and wound healing.

**Video 1.** **Visualization of Ai6 *Cass4*** ^**WT**^ **microglia reacting to a laser injury (injury location is defined by the white circle at the beginning of the video) for 40 min.** Scale bar: 20 μm. Data are representative of five independent mice analyzed.

**Video 2.** **Visualization of Ai6 *Cass4*** ^**ΔMG**^ **microglia reacting to a laser injury (injury location is defined by the white circle at the beginning of the video) for 40 min.** Scale bar: 20 μm. Data are representative of six independent mice analyzed.

## Discussion

The *CASS4* gene locus has consistently been identified in GWAS studies to be associated with an increased risk of developing AD (Lambert et al., 2013; Lin et al., 2017; Ramos de Matos et al., 2018; Schwartzentruber et al., 2021; Wang et al., 2015b). However, with a lack of basic knowledge on how CASS4 functions in the brain, our understanding of its contribution to neurodegenerative disease pathogenesis remains limited. In our studies, we revealed that loss of *Cass4* led to worsened Aβ plaque pathology and altered microglial responses. Microglia are known to migrate to Aβ pathology and form stable, protective barriers around them (Condello et al., 2015). They can also use their highly dynamic and mobile processes to compact Aβ fibrils into tightly packed aggregates, reducing Aβ aggregate surface area and thereby limiting their potential neurotoxic effects on nearby neurons (Casali et al., 2020; Condello et al., 2015; Zhao et al., 2017). Our studies highlight a novel role for CASS4 in regulating the ability of microglia to contain and encapsulate Aβ plaques. This was most pronounced in our studies, showing poor plaque compaction as well as decreased recruitment of IBA1^+^ processes to Aβ pathology in CASS4-deficient 5xFAD mice.

Our studies also suggest that CASS4 is involved in regulating focal adhesion signaling and related processes such as wound healing and microglial mobilization to injury. Interestingly, previous work has uncovered that multiple canonical focal adhesion signaling molecules are actively engaged in the AD brain. For instance, dynamic FAK signaling is commonly seen surrounding Aβ plaques in human AD patients (Grace and Busciglio, 2003; Saleh et al., 2022), and integrins have been shown to bind Aβ and activate focal adhesion signaling (Caltagarone et al., 2007). Moreover, mutations in the focal adhesion gene *PTK2B*, which encodes the protein PYK2 and is a suggested CASS4-binding partner (Barrett et al., 2013; Beck et al., 2014), have also been linked to increased AD risk in GWAS studies (Lambert et al., 2013; Lin et al., 2017; Ramos de Matos et al., 2018; Schwartzentruber et al., 2021; Wang et al., 2015b). These collective studies, coupled with the CASS4 findings presented in this manuscript, help to identify focal adhesion biology as an important player in AD. However, continued efforts are still needed to explore the role of focal adhesion signaling in AD and microglia, as the contributions of focal adhesion biology in neurodegenerative disease remain relatively understudied, especially in comparison with the recent advancements made in defining roles for innate immune pattern recognition receptors (e.g., TREM2, CLEC7A) and cytokine signaling (e.g., IL-1, type I IFN) in AD (Keren-Shaul et al., 2017; Mangalmurti et al., 2025; McQuade et al., 2020; Wang et al., 2016; Yoo et al., 2023).

M-CSF and osteopontin have also been linked to AD and focal adhesion signaling. For instance, soluble CSF1 receptor has recently been shown to enhance microglial clustering around Aβ plaques and attenuate neuronal dystrophy in 5xFAD mice (Zhang et al., 2025). Furthermore, work focused on another AD risk gene involved in focal adhesion signaling, *CD2AP*, demonstrated that CD2AP interacts with the CSF1 receptor to regulate microgliosis in 5xFAD mice (Zhang et al., 2024). Additionally, osteopontin has been suggested to have a bidirectional role in neurodegeneration, such as inducing harmful proinflammatory cascades in some contexts while mediating neuroprotective Aβ clearance in others (Azizan et al., 2025). Our studies show that loss of CASS4 impacts focal adhesion protein phosphorylation levels and disrupts M-CSF- and osteopontin-associated wound healing responses in scratch assays. Moreover, our findings suggest that CASS4 is centrally involved in coordinating in vivo microglial mobilization to laser injury, thus implicating a central role for CASS4 in these focal adhesion-related processes.

Our studies also revealed that loss of CASS4 in 5xFAD mice causes defective cognitive flexibility and exacerbated neuritic dystrophy. In line with this, snRNA-seq analysis in these mice further uncovered that this neuropathology and cognitive dysfunction were associated with an upregulation of numerous genes that have recently been linked to worsened neuronal health and neurodegenerative disease, such as *Mid1*, *Gpr88*, *Crhr1*, *Sema5b,* and downregulation of *Sv2b*, *Vav3*, *Arhgap24*, *Asap2*, *Elmo1*, and *Nrn1* (Agrawal et al., 2026; Chan et al., 2020; Cowan et al., 2005; Falace et al., 2024; Galet et al., 2021; Hale et al., 2011; Heinz et al., 2021; Kim et al., 2011; Li et al., 2018; Liu et al., 2014; Lv et al., 2024; Ma et al., 2025; Matthes et al., 2018; Meirsman et al., 2016; Miyamoto et al., 2020; O'Connor et al., 2009; Pugh et al., 2026; Sauzeau et al., 2010; Stout et al., 2019; Yoon et al., 2025). Of the brain-resident cell types, *Cass4* appears to be expressed only by microglia (Lee et al., 2023; Podlesny-Drabiniok et al., 2020; Zhang et al., 2014). Therefore, CASS4 is unlikely to be impacting neural health through cell-intrinsic roles in neurons. Instead, the increased Aβ load and defective plaque compaction seen in 5xFAD *Cass4*^−/−^ mice are more likely to be contributing to a brain environment that promotes neuronal pathology and dysfunction.

While the findings presented here break new ground in our understanding of how CASS4 impacts AD-related amyloidosis, microglial biology, and focal adhesion signaling, many questions remain regarding the underlying biology of CASS4 in AD. Most notably, future studies are needed to explore how individual human AD-associated *CASS4* gene variants affect neuroinflammation and degeneration. Although numerous single-nucleotide polymorphisms (SNPs) in the *CASS4* locus have been linked to AD (Beck et al., 2014; Bellenguez et al., 2022; EADB et al., 2026; Lambert et al., 2013; Lin et al., 2017; Schwartzentruber et al., 2021), little is currently known about how individual disease-associated mutations impact CASS4 function. Therefore, to gain initial insights into the overarching role of CASS4 in AD-related pathogenesis and microglial biology, we first generated novel germline and conditional knockout mouse models. However, future efforts leveraging discrete human AD-associated *CASS4* SNPs and gain-of-function mutations are needed to obtain a more complete understanding of CASS4 biology and its role in AD.

Despite numerous GWAS studies indicating that *CASS4* is a prominent genetic risk factor for late-onset AD, the role of CASS4 in the brain remains relatively unexplored. Here, we show that loss of *Cass4* in the 5xFAD mouse model of AD leads to worsened Aβ pathology, exacerbated neuronal dystrophy, and defective cognitive flexibility. While the absence of CASS4 did not alter microglial numbers or their ability to take on a DAM phenotype, we found that CASS4-deficient microglia display defective mobilization to Aβ plaques, which leads to poor Aβ plaque compaction and the generation of more toxic conformations of Aβ aggregates. Our work has also identified CASS4 as a pivotal regulator of focal adhesion signaling and related processes such as wound healing and microglial mobilization to injury and neuropathology. These collective findings shed new light on the role of CASS4 in neurobiology and neurodegenerative disease pathogenesis, and further suggest that targeting CASS4 may provide a potential therapeutic strategy to treat AD.

## Materials and methods

### Mice

All experiments were conducted in accordance with the guidelines and regulations of the University of Virginia and approved by the University of Virginia Animal Care and Use Committee. 5xFAD (strain #034848), *Cx3cr1*^ERT2Cre^ knock-in/knock-out (strain #021160), and Ai6 (strain #007906) mice were obtained from the Jackson Laboratory. *Cass4*^*−/−*^ and *Cass4*^fl/fl^ mice were generated via CRISPR/Cas9 gene targeting on a C57BL/6J background in collaboration with Cyagen Biosciences. *Cass4*^−/−^ mice were generated through a 354–base pair deletion in exon 2. The targeting strategy is shown in Fig. S1, A and B. 5xFAD and *Cass4*^−/−^ animals were crossed to generate 5xFAD^−/−^*Cass4*^+/+^ (WT), 5xFAD^−/−^*Cass4*^−/−^ (*Cass4*^−/−^), 5xFAD *Cass4*^*+/+*^ (5xFAD), and 5xFAD^+/−^*Cass4*^−/−^ (5xFAD *Cass4*^−/−^) mice. Recent evidence showed that the parental origin of the 5xFAD transgene impacts Aβ burden in offspring (Sasmita et al., 2025); thus, all mice inherited the 5xFAD transgene from the male parent. Cohorts in our studies consisted of only female mice, given that amyloid pathology is more accelerated and consistent in female 5xFAD mice compared with male 5xFAD mice (Forner et al., 2021; Sasmita et al., 2025). *Cass4*^fl/fl^ mice were generated with a gRNA targeting the *Cass4* gene and a donor vector containing loxP sites flanking exon 1. The targeting strategy is shown in Fig. S4, E and F. For in vivo studies, Ai6, *Cx3cr1*^ERT2Cre^, and *Cass4*^fl/fl^ animals were crossed to generate Ai6^+/−^*Cass4*^*+/+*^*Cx3cr1*^ERT2Cre^ (Ai6 *Cass4*^WT^) and Ai6^+/−^*Cass4*^fl/fl^*Cx3cr1*^ERT2Cre^ (Ai6 *Cass4*^ΔMG^) mice. To induce Cre recombinase-mediated excision of loxP-flanked DNA sequences, mice were fed tamoxifen chow for 2 wk following weaning. Mice were then returned to standard chow for the remainder of the study. All mice were housed with food and water available ad libitum under specific pathogen–free conditions with controlled temperature (21 ± 1.5°C) and humidity (50 ± 10%) and subjected to the standard 12-h light/dark cycle.

### Brain sample preparation

Brain samples were collected and processed as previously described (Ennerfelt et al., 2022). Mice were euthanized using CO_2_ asphyxiation and transcardially perfused with 20 ml of ice-cold 1× PBS (J67802.K2; Thermo Fisher Scientific). Brains were dissected out, and the cerebellum and olfactory bulbs were removed. The left hemisphere was drop-fixed in 4% paraformaldehyde (PFA) (15714-S; Electron Microscopy Sciences) overnight and then transferred to 30% sucrose (S0389; Sigma-Aldrich) for 48 h at 4°C. The right hemisphere was flash-frozen on dry ice and stored at −80°C. Drop-fixed samples were frozen in Tissue-Plus OCT (4548; Sakura) on dry ice, and sagittal sections 50 μm in thickness were cut using a cryostat (CM1950; Leica). Tissue sections were stored at 4°C in 1× PBS and 0.05% sodium azide (71448-16; Ricca Chemical) for downstream staining and imaging. Flash-frozen hemispheres were thawed for protein extraction and mechanically homogenized in 250 μl of Tissue Protein Extraction Reagent (T-PER, 78-510; Thermo Fisher Scientific) containing phosphatase inhibitor PhosSTOP (04-906-845-001; Roche) and protease inhibitor cOmplete (11-873-580-001; Roche). For snRNA-seq experiments, the hippocampus was microdissected from both hemispheres, flash-frozen in liquid nitrogen, and stored at −80°C.

### Quantitative PCR (qPCR)

TRIzol (15596-018; Thermo Fisher Scientific) was added to 50 μl of homogenized brain before adding 200 μl chloroform (BP1145-1; Thermo Fisher Scientific). Samples were incubated at room temperature for 5 min and spun down at 14,000 rpm at 4°C for 15 min. The top fraction was collected and incubated with an equal volume of isopropanol (I9516; Sigma-Aldrich) for 10 min at room temperature. The samples were spun down at 12,000 rpm at 4°C for 10 min, followed by two washes with 70% ethanol (459828; Sigma-Aldrich). After the last wash, ethanol was aspirated off, and the pellet was left to air-dry for 15 min before adding 30 μl DNase/RNase free water (10977023; Thermo Fisher Scientific). Sample quality was assessed using the NanoDrop 2000 Spectrophotometer (Thermo Fisher Scientific). cDNA synthesis was achieved using SensiFast cDNA Synthesis kits (BIO-65054; BioLine). Expression levels of *Cass4* (Mm07292046_m1) were determined using the SensiFast Probe No-ROX kit (BIO-86005; BioLine) and the CFX384 real-time PCR machine (1855484; Bio-Rad) following the manufacturer’s protocols.

### Immunofluorescence staining and microscopy

Brain sections were washed in 1× PBS three times for 5 min at room temperature before being blocked with 2% donkey serum (S30-100ML; Sigma-Aldrich), 1% BSA (BP1600-100; Thermo Fisher Scientific), 0.1% Triton-X (X100-1L; Sigma-Aldrich), and 0.05% Tween-20 (P1379-500ML; Sigma-Aldrich) in 1× PBS for 2 h at room temperature while rocking. When mouse primary antibodies were used, M.O.M. IgG Blocking Reagent (MKB-2213; Vector Laboratories) was used according to the manufacturer’s instructions. Primary antibody mixes, diluted in block solution, were applied overnight at 4°C while rocking. The following primary antibodies were used: anti-IBA1 (ab5067; Abcam, 1:300), anti-CD68 (MCA1957; Bio-Rad, 1:1,000), anti-APP (ab326; Abcam, 1:750), anti-ATG9A (ab108338; Abcam, 1:500), anti-β amyloid, 1-16 (803014; BioLegend, 1:1,000), anti-PU.1 (MAB7124; R&D Systems, 1:300), anti-GFAP (130300; Thermo Fisher Scientific, 1:1,000), anti-P2RY12 (ab300141; Abcam, 1:1,000), anti-AXL (AF854; R&D Systems, 1:50), and anti-GAL3 (125408; BioLegend, 1:500). Sections were washed with wash buffer (1× PBS and 0.05% Tween-20) three times for 5 min at room temperature and stained with matching Alexa Fluor 488, 594, or 647 donkey anti-rabbit, -goat, -mouse, or -rat (Thermo Fisher Scientific, 1:1,000) secondary antibodies at room temperature for 2 h while rocking. Sections were further stained with Thioflavin S (0.2 mg/ml, T1892; Sigma-Aldrich) for 8 min in 50% ethanol while rocking and were washed three times for 2 min in 50% ethanol and two 2-min washes with 1× PBS at room temperature. All sections were then transferred to fresh 1× PBS prior to being mounted on glass slides (12-550-15; Fisherbrand) with ProLong Gold antifade reagent (P36930; Invitrogen) and coverslips (12-544C; Fisherbrand). Mounted slides were stored at 4°C protected from light, and imaged on a Leica Stellaris 5 confocal microscope using LAS AF software (Leica Microsystems). All confocal images were acquired in 8-bit format at 2,048 × 2,048 pixel resolution with no zoom. For all imaging experiments, three brain sections per mouse were imaged, with at least three FOV taken per section per region (a total of 9–12 images per mouse). Quantification of images (such as counts, volumes, sphericity, and intensity) was performed using ImageJ 1.54p software or IMARIS 10 software.

### MWM

All behavior experiments were performed between 8 a.m. and 12 p.m. in a blinded fashion. All mice were 4 mo old at the time of the assay. Mice were transported from their home vivarium room to the behavior core and allowed 30 min to habituate at the beginning of each day of behavior testing, and then transported back to their home vivarium room at the end of each testing day. All testing was tracked and scored using EthoVision XT video tracking software (Noldus). Mice were gently placed in a 19–22°C pool filled with white paint and a hidden platform 1 cm below the opaque water level. The pool contained four different visual cues with varying shapes and colors, and mice were placed in alternate places in the pool for each trial. For the first 4 days, mice were trained on the location of a hidden platform location 1). Each day consisted of four 60-s trials. If the mouse found the platform within the 60 s, the mouse was removed from the pool. If the mouse failed to find the platform after 60 s, the mouse was guided to the platform and then removed from the pool after remaining on the platform for 8 s. On the fifth day, the platform was removed, and a memory probe for location 1 was performed. On the sixth day, the platform was moved to the opposite quadrant of the pool and mice were retrained for 4 more days on the new location (location 2). After the ninth day, the platform was again removed, and a memory probe for location 2 was performed. Upon completion of the second probe, visible control trials were performed by moving the platform to a new location and denoting its location with a visible marker. Mice were given three 60-s trials to find the platform. Any mouse that did not find the platform after these trials was excluded.

### Open field

All mice were individually placed in the top left corner of a white Plexiglass-walled chamber (40 × 40 cm^2^) and allowed to explore the arena for 10 min.

### Elevated plus maze

Mice were placed in the center square of a maze that has two open arms (35 × 6 cm^2^) and two closed arms (35 × 6 cm^2^) with 20-cm-tall black plexiglass walls. Mice were allowed to explore during a 5-min trial.

### In vivo Aβ phagocytosis assay

The in vivo Aβ phagocytosis assay was performed as described in Ennerfelt et al. (2022). Briefly, 5-mo-old 5xFAD *Cass4*^−/−^ and 5xFAD littermate controls were intraperitoneally injected with 10 mg/kg methoxy-X04 (B5769; ApexBio) in a 1:1 ratio of PBS and DMSO (D2650; Sigma-Aldrich). 3 h after injection, euthanasia of mice was performed according to the University of Virginia Animal Care and Use Committee using CO_2_ followed by transcardial perfusion using 20 ml of ice-cold 1× PBS. The meninges, cerebellum, and olfactory bulbs were removed from the brain before placing brains in 5 ml HBSS (14025092; Gibco) with Mg and Ca, 4 U/ml papain (LS003126; Worthington), and 50 U/ml DNase I (10104159001; Sigma-Aldrich). Following three triturations of the samples over 45 min at 37°C, the brain homogenates were transferred to a conical tube containing a 70-μm cell strainer (229483; CELLTREAT) and topped with 20 ml DMEM/F12 (21331020; Gibco) containing 10% FBS (FBS-01-0500; Rockland), 1× antibiotic-antimycotic (15240096; Thermo Fisher Scientific), and 1× GlutaMAX (35050061; Invitrogen). Strained samples were then spun down with slow brake (3 on a 0–10 scale) for 10 min at 300 g before being resuspended in 13 ml of 37% Percoll (Cytvia; 17-0891-02) and spun down with no brake at 2,000 rpm for 12 min. Myelin was removed, and cells were resuspended in FACS buffer (pH 7.4; 0.1 M PBS; 1 mM EDTA, and 1% BSA). Flow cytometry for microglia was then performed (as described below) with additional gating for methoxy-X04.

### Flow cytometry staining and analysis

Following generation of a single-cell suspension, cells were plated in a 96-well plate and incubated for 10 min in 50 μl Fc block (1 μg/ml 2.4G2 Ab [70-0161-M001; BioXCell], 0.1% rat γ-globulin [012-000-002; Jackson ImmunoResearch]) at room temperature. For extracellular staining, cells were incubated with eBioscience Fixable Viability Dye eFluor780 (65-0865-14; Thermo Fisher Scientific, 1:800), as well as primary anti-mouse antibodies diluted in FACS buffer: CD45-PeCy7 (25-0451-82; Thermo Fisher Scientific), CD11b AF700 (56-0112-82; Thermo Fisher Scientific), and Ly6C BV605 (128035; BioLegend) at a concentration of 1:200, for 30 min at 4°C. Cells were then washed and permeabilized/fixed overnight at 4°C with Foxp3/Transcription Factor Fixation/Permeabilization Buffer (00-5521-00; Thermo Fisher Scientific). For intracellular staining, cells were then incubated with primary anti-mouse antibodies diluted in permeabilization buffer: CD68-PE (12-0681-82; Thermo Fisher Scientific, 1:200) and Ki67 eFluor506 (69-5698-82; Thermo Fisher Scientific, 1:100) for 30 min at 4°C. Cells were then washed with FACS buffer and acquired on a Cytek Aurora 5-laser flow cytometer (5L, UV-V-B-YG-R) using the SpectroFlo software (version 3.3.0, Cytek Biosciences). Flow cytometry data were gated and analyzed using FlowJo (version 10.10.1). The gating strategy was performed as follows: (1) SSC-H versus FSC-H to exclude debris; (2) PE-Cy7 versus time to exclude any flow rate discrepancies; (3) SSC-A versus SSC-H to select singlets; (4) SSC-A versus Live/Dead for dead cell exclusion; (5) SSC-A versus CD45 to select CD45^+^ leukocytes; (6) CD11b versus Ly6C within CD45^int^ leukocytes to select brain macrophages; (7a) CD11b versus Ki67 within brain macrophages to select the proliferative subset; (7b) SSC-A versus CD68 within brain macrophages to select the phagocytic subset; (8) SSC-A versus methoxy-X04 within the phagocytic subset to select the Aβ phagocytic population.

### MACS isolation of macrophages and phospho-array

Euthanasia of mice was performed according to the University of Virginia Animal Care and Use Committee using CO_2_ followed by transcardial perfusion using 20 ml of ice-cold 1× PBS. The meninges, cerebellum, and olfactory bulbs were removed, and brains were placed in 5 ml HBSS (14025092; Gibco) with Mg and Ca, with 4 U/ml papain (LS003126; Worthington) and 50 U/ml DNase I (10104159001; Sigma-Aldrich). Following three triturations of the samples over 45 min at 37°C, the brain homogenates were transferred to a conical tube containing a 70-μm cell strainer and topped with 20 ml DMEM/F12 (21331020; Gibco) containing 10% FBS (FBS-01-0500; Rockland), 1× antibiotic-antimycotic (15240096; Thermo Fisher Scientific), and 1× GlutaMAX (35050061; Invitrogen). Strained samples were then spun down with slow brake (3 on a 0–10 scale) for 10 min at 300 *g*, resuspended in 160 μl MACS buffer (130-091-376; Miltenyi Biotec), and then incubated with 20 μl MACS CD11b (microglia) microbeads (130-093-634; Miltenyi Biotec) for 15 min at 4°C. Sorting was performed using LS columns and a QuadroMACS magnet (130-042-401, 130-091-051; Miltenyi) according to the product instructions. Cell pellets were obtained by spinning at 300 *g* for 5 min at 4°C and then frozen at −80°C until they were submitted to Full Moon BioSystems. The Antibody Array Assay Service was used to perform the Phospho Explorer Antibody Array (PEX100; Full Moon Biosciences). Two separate assays were performed on a total of five mice per genotype, and the results were averaged across both assays. Final lists of differentially phosphorylated proteins included all proteins that had an average log fold change >1.4 or <−0.4.

### Primary cell culture

Primary microglia cells were prepared from postnatal day 0–3 pups of WT and *Cass4*^−/−^ mice as previously described (Lee et al., 2023). Briefly, brains were surgically excised, and meninges were removed from cortical tissue in ice-cold dissection buffer (HBSS supplemented with 1% HEPES [15630080; Thermo Fisher Scientific], 1 M sodium pyruvate [11360-070; Gibco], and 1% penicillin/streptomycin [15070063; Thermo Fisher Scientific]). After dissection, isolated cortices were stored in a Petri dish on ice containing growth medium DMEM-F12 (21331020; Thermo Fisher Scientific), 10% HI- FBS, and 1% penicillin/streptomycin. Tissue from four to five mixed sex pups of the same genotype was pooled. Cortices were finely minced and then transferred to a 15 ml conical tube and dissociated with 5 ml 0.25% trypsin-EDTA (25200056; Thermo Fisher Scientific) for 25 min in a 37°C water bath with gentle agitation. An equal amount of growth medium was added to neutralize the trypsin, and the tubes were centrifuged at 300 *g* for 5 min. After removing the supernatant, the tissue was washed three times with 2 ml of warm HBSS. The tissue was then triturated in 10 ml of warm growth medium and passed through a 70-μm cell strainer to remove large particulates. Warm growth medium was added to a final volume of 10 ml per mouse brain collected and seeded in T75 flasks (658175; VWR) incubated at 5% CO_2_ and 37°C. Medium was replaced with fresh growth medium after 24 h. At day 7, medium was replaced with fresh growth medium supplemented with 50 ng/ml M-CSF (315-02; PeproTech). Peak microglial confluence in the primary mixed glial cultures typically occurred around day 12–14, at which point the flasks were shaken at 240 rpm for 2 h at 37°C (88881101; Thermo Fisher Scientific). Supernatant containing detached microglia was pooled and centrifuged at 300 *g* for 5 min. Cells were then resuspended and plated for experiments. All experiments were performed within 2–4 days of plating. Purity of primary microglia cultures was authenticated by immunocytochemistry using anti-IBA1 (234-308; Synaptic Systems, 1:1,000), anti-GFAP (2.2B10; Thermo Fisher Scientific, 1:1,000), and anti-OLIG2 (AB9610; Millipore Sigma, 1:1,000).

### Immunocytochemistry

Primary microglia cells were plated onto Poly-D-Lysine and laminin-coated coverslips (354087; Corning) and allowed to adhere overnight. Cells were then cultured in either plain growth media or in growth media supplemented with 50 ng/ml M-CSF (315-02; PeproTech) for 6 h. Media was then removed, and coverslips were washed three times with PBS before adding 4% PFA and incubating at room temperature for 15 min. Cells were then washed three times with PBS and blocked at room temperature for 1 h with 5% HI-FBS and 3% Triton X (X100-1L; Sigma-Aldrich) in PBS. Anti-PAXILLIN (AF4259; R&D Systems, 10 μg) was added overnight in 1% HI-FBS and 0.1% Triton X at 4°C. Coverslips were washed three times with PBS and then incubated, covered, for 1 h at room temperature with matching Alexa Fluor 488 donkey anti-sheep (Thermo Fisher Scientific, 1:1,000) secondary antibodies in 5% HI-FBS and 3% Triton X (X100-1L; Sigma-Aldrich) in PBS. After washing three times with PBS, coverslips were counterstained with DAPI (D9542-5MG; Sigma-Aldrich, 1:1,000) for 10 min, covered, at room temperature, before being mounted onto glass slides (12-550-15; Fisherbrand) with ProLong Gold antifade reagent (P36930; Invitrogen). Mounted slides were stored at 4°C, protected from light, and imaged on a Leica Stellaris 8 confocal microscope using LAS AF software (Leica Microsystems). All confocal images were acquired in eight-bit format at 1,024 × 1,024-pixel resolution at 63× magnification. For paxillin imaging experiments, two technical replicates were plated, and 12 FOVs were imaged per experiment. Three independent experiments were performed, with a total of 70–80 images taken per group. Quantification of surface area was performed using ImageJ 1.54p software.

### BMDM culture and scratch assay

BMDMs were isolated and cultured as described previously (Ennerfelt et al., 2022). Briefly, bone marrow was extracted from the tibia and femurs of WT and *Cass4*^−/−^ mice using a benchtop centrifuge. To lyse red blood cells, 1 ml of ACK lysis buffer (A1049201; Thermo Fisher Scientific) was added to the isolated marrow for 5 min. The remaining cells were passed through a 70-μm cell strainer and then plated into 10-cm dishes in BMDM media (RPMI 1640,11875119; Thermo Fisher Scientific, 10% FBS, and 1% antibiotic-antimycotic). Cultures were supplemented with 50 ng/ml M-CSF (312-02; PeproTech) for the first 6 days, with a media change at day 3. Mature cells were plated for experiments on days 6–8 after harvest in BMDM media. For scratch wound assays, cells were plated in three technical replicates to confluency in 24-well plates and allowed to adhere overnight. The next day, a scratch wound was gently made in the cell layer using a p200 pipet tip. Wells were washed three times with 1× PBS before treatment media (50 ng/ml M-CSF, 400 ng/ml osteopontin [441-OP; R&D Systems], or 2 μM Cytochalasin D [C8273; Sigma-Aldrich]) were added. Plates were immediately placed into an Incucyte SX5 (Sartorius) and imaged every hour for 24 h. Wound closure was assessed using the Incucyte Cell-by-Cell module and normalized to the starting wound size of each well.

### snRNA-seq

Flash-frozen hippocampi from six mice per genotype were pooled for snRNA-seq and submitted to GENEWIZ (Azenta Life Sciences). Nuclei extraction, sequencing, and analysis were performed as described previously (Samuels et al., 2023), but with a target capture of at least 10,000 Gel Beads-in-Emulsion. Raw feature matrices containing the barcodes, features, and matrix files for each sample were used for further analysis in RStudio. The Seurat package in RStudio was used for downstream analyses. The SoupX package was run to reduce ambient RNA contamination. For quality control, nuclei containing a low (<200) or high number (>8,500) of detected genes, >1% mitochondrial reads, and >5% ribosomal genes were removed. Doublet nuclei were further filtered out using scDblFinder. After filtering, 33,985 nuclei remained. Log-normalization, scaling, and integration were then applied. Principal component (PC) analysis was conducted using the top 3,000 most variable features, and UMAP reduction was conducted based on the top 15 PCs as determined by an ElbowPlot. The Harmony algorithm was used to correct for batch effects. Clustering was performed using the FindNeighbors and FindClusters functions. A resolution of 0.1 was chosen for downstream clustering. The identification of each cluster was determined using a comprehensive list of known marker genes for brain cell types. Following clustering, nuclei populations were visualized in 10 distinct clusters, including inhibitory neurons, excitatory neurons, microglia, astrocytes, endothelial cells, pericytes, oligodendrocytes, and oligodendrocyte precursors. One small mixed glia cluster consisting of 148 total nuclei was removed from our analysis. Nuclei from the microglia and excitatory neuron clusters were isolated to further analyze specific subpopulations. Microglia and neurons were reintegrated and then analyzed using the steps outlined above. The top five PCs and a resolution of 0.1 were chosen for conducting the excitatory neuron UMAP, whereas the top 10 PCs and a resolution of 0.3 were chosen for conducting the microglia UMAP analysis. All UMAP, dot plot, and heatmap visualizations were performed using Seurat and ggplot2. Differential expression analysis between conditions was conducted using the FindMarkers function in Seurat and the MAST algorithm. To minimize RNA artifacts, mitochondrial genes (-*mt*), ribosome genes (-*rpl*, -*rps*), unannotated genes (-*gm*, *-rik$*, *-ac*, and -*loc*), and pseudogenes (-*ps$*) were excluded from all clusters, and genes canonically restricted to neurons (such as *Kcnip4* and *Cadm1*) were excluded from glial cell clusters. For visualization, volcano plots were created using the EnhancedVolcano package in R, and DEGs with adjusted P values of <0.05 and average log_2_foldchange >0.1 or <−0.1 were plotted. Final lists of DEGs containing all genes with adjusted P values of <0.05 and average log_2_foldchange >0.25 or <−0.25 were used for downstream pathway analysis. Pathway analysis was performed using the Enrichr tool to evaluate specific pathway databases, including GO Cellular Component 2025, GO Biological Process 2025, GO Molecular Function 2025, and Mouse WikiPathways 2024. The Combined Score was reported, which integrates statistical significance (Fisher exact test P value) and enrichment magnitude (z-score) to prioritize biologically relevant pathways (Chen et al., 2013). The code used for this study is available on GitHub (https://github.com/LukensLab) and upon reasonable request from the corresponding author.

### Cranial window surgery

To implant mice with cranial windows, surgical plane anesthesia was achieved using 2–5% isoflurane. Bupivacaine was injected at the site of surgery prior to incision. Hair was then removed by application of Nair; the surgical site was cleaned using three alternating rounds of betadine and ethanol, with subsequent removal of the skin. The periosteum was then removed using a sterile scalpel blade, followed by gentle scoring of the skull. A 3 mm No. 1 cover glass (64-0720; Warner Instruments CS-3R) was then implanted and centered 2 mm posterior and 3 mm lateral to bregma. This was then glued to the skull using Krazy all-purpose super glue plus, affixed, and sealed with C&B Metabond dental cement (S380; Parkell). Buprenorphine SR was then administered peritoneally at the end of surgery. All mice were allowed to recover 14 days before experiments commenced.

### Laser-injury image analysis and acquisition

An Olympus Dual Beam FVMPE-RS multiphoton microscope, with a MaiTai HP DS-OL pulsed laser, was utilized. Two cooled GaAsP and two multi-alkali photomultiplier detectors allowed for simultaneous 4-channel multiplexing, and an XLPLN25X/1.05 NA water-immersion objective (Olympus) was used for image acquisition. Barrier filters used included Olympus FV30-FVG (blue light at 410–455 nm and green light at 495–540 nm; dichroic at 475 nm) and FV30-FRCY5 (red light at 610/70 and far red at 705/90; dichroic at 650 nm). The FV30-SDM570 dichroic mirror was utilized to split light <570 nm to the RNDD3/4 detector and light >570 nm to the RNDD1/2 detectors. To image, animals were placed on a Kopf stereotax with a heating pad and were anaesthetized with isoflurane to ∼100 beats per minute, with their vitals being constantly monitored. Imaging was performed 100–200 µm below the surface of the somatosensory cortex. To keep anesthesia as low as possible, isoflurane anesthesia was initially set very low after induction. Upon the mouse moving its hind legs, anesthesia was then increased until this movement stopped. Only then did imaging for the experiment commence. A 40-min zoom 3 pre-injury baseline image was recorded in high-low mode. To ensure consistent thresholding, one cell was chosen as a reference point for oversaturation. Throughout the t series acquisition, laser power was adjusted such that this location maintained the same histogram level of oversaturation, which equated to one very small location in the center of the chosen soma. Once baseline imaging was complete, a laser injury was performed by increasing to zoom 5 and utilizing a circular ROI between cells of interest to induce injury. The injury was performed at 25% laser power and a pixel dwell time of 100 μs/pixel for 20 s. If this failed, the laser power was increased by 2%. Following a check that we still had a sufficient water lens, we would then repeat the same process in the postinjury t series acquisition as in the baseline image acquisition. Nikon Elements was used to measure the area and fluorescent change in Ai6^+^ cells surrounding the injury 40 min following laser injury compared with microglial area change 40 min before the injury. To do this, .oir files were first converted to sum intensity projections with ImageJ software and saved as .tiff files. These were then opened in Elements, with pixel-to-micron ratio and step size set accordingly, and then saved as a .nds file. Based on well-annotated images at the time of acquisition, the rotating rectangle feature was used to separate out the region of microglia surrounding the location of laser injury. This feature allows separation of relevant locations without changing the underlying metadata. A threshold was then set for every sum projection image in the t series, and the area of Ai6^+^ cells was recorded. Max projection representative images and videos were made with ImageJ software.

### Statistical analysis

Mean values, SEM values, unpaired Welch’s *t* tests, and two-way ANOVA were calculated using Prism software (GraphPad). Nested data for immunohistochemistry were analyzed by a linear mixed-effects model using the lme4 package in R. P values <0.05 were considered significant. Outliers were identified and excluded using the Robust regression and OUTlier removal method in GraphPad Prism 11, with the false discovery rate set to 1%.

### Online supplemental material

We present five supplemental figures and two supplemental videos. Fig. S1 shows validation of *Cass4* deletion and clustering from the snRNA-seq experiment. It also shows a subcluster analysis of the excitatory neuron population. Fig. S2 shows results from the MWM, open field, and elevated plus maze in 5xFAD and 5xFAD *Cass4*^−/−^ mice and in WT and *Cass4*^*−/−*^ mice. Fig. S3 shows cortical data for 5xFAD and 5xFAD *Cass4*^−/−^ mice, the gating strategy for the methoxy-X04 study, its results for Ki67 and methoxy+ CD68^+^ microglia, as well as results from immunohistochemical staining for GAL3 and CD68. It also shows snRNA-seq analysis of the microglia population. Fig. S4 shows immunohistochemical staining for microglial markers in WT and *Cass4*^*−/−*^ mice, as well as validation of microglia-targeted *Cass4* deletion in the *Cass4*^ΔMG^ mice. Lastly, Fig. S5 shows all differentially phosphorylated proteins identified in the phosphoproteomic array and scratch assays performed with osteopontin-treated BMDMs. Video 1 shows the microglial response to laser injury in Ai6 *Cass4*^WT^ microglia. Video 2 shows the microglial response to laser injury in Ai6 *Cass4*^ΔMG^ microglia.

## Data Availability

The data underlying the figures are available in the published article and its online supplemental material. Single-nucleus sequencing data have been deposited in the GEO repository public website (accession number GSE348326). Source data have been uploaded to Figshare at the following DOI: 10.6084/m9.figshare.34238421.
